# Understanding the tonifying and the detoxifying properties of Chinese medicines from their impacts on gut microbiota and host metabolism: a case study with four medicinal herbs in experimental colitis rat model

**DOI:** 10.1186/s13020-022-00673-w

**Published:** 2022-10-04

**Authors:** Ting Li, Xuejiao Gao, Zhixiang Yan, Tai-Seng Wai, Wei Yang, Junru Chen, Ru Yan

**Affiliations:** 1 State Key Laboratory of Quality Research in Chinese Medicine, Institute of Chinese Medical Sciences, University of Macau, Taipa, Macao China; 2 Zhuhai UM Science & Technology Research Institute, Zhuhai, 519080 China

**Keywords:** Chinese medicines, Tonic herb, Detoxifying herb, Experimental colitis, Gut microbiota, Host metabolism

## Abstract

**Background:**

Chinese medicines (CMs) have emerged as an alternative therapy for ulcerative colitis through reinforcing the vital *qi* and/or eliminating the pathogenic factors according to the traditional Chinese medicinal theory. Presystemic interactions of CMs with gut microbiota and the associated metabolic network shift are believed to be essential to achieve their holistic health benefits in traditional oral application.

**Methods:**

This study first employed 16S rDNA-based microbial profiling and mass spectrometry-based urinary metabolomics to simultaneously evaluate four single CMs frequently prescribed as main constituent herbs for alleviating UC, the tonic ginseng and Astragali Radix (AR) and the detoxifying Scutellaria Radix (SR) and Rhubarb, on a dextran sodium sulfate (DSS)-induced colitis rat model, with aims to understanding the tonifying or detoxifying properties of CMs through clinical phenotypes, the common features and herb-specific signatures in gut microbial alterations and the associated host metabolic shifts. Colitis was induced in rats receiving 5% DSS for consecutive 7 days. Control group received water alone. Herbal groups received 5% DSS and respective herbal preparation by gavage once daily. Body weight, stool consistency, and rectal bleeding were recorded daily. Feces and urine were freshly collected at multiple time points. On day 7, blood and colon tissues were collected to determine anti-/pro-inflammatory cytokines levels, colonic myeloperoxidase activity, and histopathologic alterations.

**Results:**

Gut microbiome was more prone to herb intervention than metabolome and displayed increasing associations with metabolic dynamics. Although both the tonic and the detoxifying herbs alleviated colitis and caused some similar changes in DSS-induced microbiome and metabolome disturbance, the tonic herbs were more effective and shared more common microbial and metabolic signatures. The detoxifying herbs elicited herb-specific changes. Rhubarb uniquely affected phenylalanine metabolism and established high correlations between *Akkermansia muciniphila* and *Parasutterella* and hydroxyphenylacetylglycine and phenylbutyrylglycine, while SR caused significant elevation of steroidal glucuronides dehydropregnenolone glucuronide and estriol glucuronide, both displaying exclusive correlations with genus *Acetatifactor*.

**Conclusion:**

Both tonic and detoxifying herbs tested ameliorated experimental colitis and elicited alternative microbial and host metabolic reprogramming. The findings highlight the importance of presystemic interactions with gut microbiota to host metabolic shifts and promote modern translation of tonic and detoxifying properties of CMs.

**Supplementary Information:**

The online version contains supplementary material available at 10.1186/s13020-022-00673-w.

## Introduction

Ulcerative colitis (UC), a subtype of inflammatory bowel disease (IBD), is a chronic intermittent disorder characterized by immune imbalance and gut dysbiosis [1]. UC therapy has become a worldwide clinical challenge because of largely unknown etiology, complex pathogenesis, increasing incidence, and poor prognosis. Multiple factors, including genetics and environmental factors, epithelial barrier dysfunction, and immunological dysregulation, are involved in the pathology of UC [1]. Microbial dysbiosis is also believed to be an essential player in UC pathogenesis which was characterized by reduced gut microbial diversity [2]. Fecal microbiota transplantation (FMT) or chemical inhibition of a specific family such as *Enterbacteriaceae* showed efficacy in dextran sulfate sodium (DSS)-induced colitis models [3, 4]. FMT from healthy donors to patients with UC improved remission in clinical trials [5]. On the other hand, metabolomics studies unraveled characteristic metabolic shifts in experimental colitis models [6], including some host-microbe co-metabolites, such as phenylacetylglycine, hippurate, and indoxyl sulfate. Also, gut metabolome in UC patients revealed some differential metabolites including bile acids and their derivatives, amino acids, phenylacetamides, as well as host-microbial cometabolites such as indoles and fatty acids, which showed robust associations with differential bacterial species including *Ruminococcus gnavus*, *Ruminococcus callidus*, *Lachnospiraceae bacterium*, etc. [7]. These findings support the involvement of gut microbiota and the contribution of gut microbial metabolism to host metabolic phenotype in UC development.

Western medicines such as aminosalicylic acid, corticosteroid, and immune suppressive drugs are most frequently prescribed for alleviating colonic inflammation, but long-term uses of these medicines cause drug resistance [8] and/or adverse reactions, such as nausea, vomiting, headache [9]. Recently, Chinese medicines (CMs) have emerged as an alternative therapy for IBD. According to the traditional Chinese medicinal (TCM) theory, IBD can be alleviated through reinforcing the vital *qi* and/or eliminating the pathogenic factors [10, 11]. As such, tonic herbs, such as ginseng (GS, *renshen* in Chinese) and Astragali Radix (AR, *huangqi* in Chinese), as well as detoxifying herbs such as Scutellariae Radix (SR, *huangqin* in Chinese) and Rhubarb (RB, *dahuang* in Chinese), have been frequently prescribed as the main constituent herbs of many compound formulas used for alleviating gastrointestinal disorders including UC. For instance, *Rhubarb-Peony* Decoction recorded in *Jin Gui Yao Lve* [12] and *Huangqin* decoction documented in *Shang Han Lun* [13], two famous Chinese medicinal monographs authored by Zhongjing Zhang nearly two millennia ago, contain RB and SR as the monarch, respectively. *Li-Zhong* decoction, a well-known formula recorded in *Shang Han Lun*, contains ginseng as the minister [14]. AR and SR are the main constituent herbs of *Qingchang*-*Huashi* formula [15]. Both these compound formulas and a variety of products of these main constituent herbs, such as extracts, fractions, or single compounds, have demonstrated therapeutic benefits in experimental colitis models [16–19]. Growing evidence indicates that the tonic nature is usually associated with immune regulatory effects [20] and the stimulation of the growth of beneficial gut bacteria [21, 22], while the detoxifying effects could be mainly ascribed to the suppression of the pathogenic bacteria [23] and/or the facilitation of the toxin excretion via metabolism such as glucuronidation [24]. However, so far, the tonic and detoxifying properties recorded in traditional medical literature are obscure, demanding a translation with modern scientific language to promote the mechanistic understanding of diseases, precise applications of CMs as well as innovative drug discovery and development. So far, the tonic and detoxifying effects of CMs have not been investigated simultaneously on UC to compare their efficacies and to find whether they share some common features and display differential profiles as well.

The multi-component and multi-target holistic nature of CMs demand systems-based approaches for understanding the molecular events underlying the health benefits observed. Given the traditional oral route in TCM practice and the chemical complexity of CMs, the presystemic interaction with gut microbiota seems inevitable and is believed to play an essential role in their therapeutic merits in chronic ailments, especially the gastrointestinal disorders, via pharmacodynamic and/or pharmacokinetic mechanisms. For instance, the interaction of gut microbiota with calycosin-7-*O*-β-d-glucoside, which is the major flavonoid component in AR but showed nil exposure in blood after oral administration to rats, stimulated the growth of the beneficial gut bacteria *Lactobacillus* and *Bifidobacterium*, while its host-microbe co-metabolite calycosin-3′-glucuronide presented as the main circulating drug-related component and exhibited a strong angiogenic effect [21]. Ginseng polysaccharides could alleviate DSS-induced colitis symptoms by reinstating the gut microbial structure and enhancing systemic exposure of co-existing small molecules ginsenosides [25]. Mori Cortex alleviated DSS-induced colitis by upregulating intestinal P-glycoprotein expression via direct and gut microbiota-mediated mechanisms to strengthen gut barrier function [26]. The rapid advancement of the multi-omics techniques as well as the multivariate data analysis approaches allows the capture of the global gut microbial alterations and host metabolic shifts in diseases and medication. There is an increasing number of applications of both microbial sequencing and metabolomics in investigations of compound formulas [14, 22, 27–29]. A few studies have systematically investigated the dynamic microbiome and metabolome disturbance and their associations in UC receiving herb interventions [15, 30, 31]. These research efforts have facilitated the translation of the TCM syndromes and holistic actions of CMs into modern scientific data and brought our understanding of the mystery of the old tradition to unprecedented depths [19, 28]. The invasiveness of blood sampling and the potential impact of blood loss on physiology and gut microbial community limit the multiple blood sampling for comprehensive metabolomics profiling. Urinary metabolomics is non-invasive, allowing multiple sampling to characterize dynamic metabolic changes without interfering with gut microbial structure and global metabolism. Additionally, metabolites generated by microbial metabolism and/or host-microbe co-metabolism enter the blood and then mainly excreted into the urine. Thus, urinary metabolomics can also unravel the contribution of gut microbial metabolism to the global host metabolic network in diseases and medications, making it more suitable than fecal metabolome to assess the impact of gut microbial changes on host metabolism.

Therefore, to understand the tonic and detoxifying properties of CMs from the viewpoints of gut microbiota and their contributions to host metabolism, this study first simultaneously investigated the representative detoxifying herbs RB and SR and tonic herbs AR and GS on a DSS-induced acute colitis rat model to compare their effects on clinical symptoms and inflammatory mediators and to characterize gut microbial changes and global metabolic shifts employing 16S rDNA-based gut microbial sequencing and LC–MS/MS-based urinary metabolomics. An association analysis was followed with attempts to identify both common and herb-specific features in gut microbiota architecture and the metabolic signatures.

## Materials and methods

### Reagents

Dextran sulfate sodium (DSS) (MW: 36,000–50,000) was purchased from MP Biomedicals (CA, USA). Enzyme-linked immunosorbent assay (ELISA) kits for measuring rat tumor necrosis factor α (TNF-α), interleukin 1β (IL-1β), interleukin 6 (IL-6), interleukin 4 (IL-4), and interleukin 10 (IL-10) were purchased from ExCell Biological Co. Ltd (Shanghai, China). Bicinchoninic acid (BCA) assay kit was supplied by Thermo Fisher Scientific Inc (Waltham, MA, USA). Myeloperoxidase (MPO) was obtained from Sigma (CA, USA). Trizol, reverse transcription kit, primers, bacterial DNA extraction kit, and SYBR Premix Ex Taq (Perfect Real-time) PCR kit were purchased from TaKaRa (Hongkong, China). Mass spectroscopy grade organic solvents acetonitrile, methanol, and formic acid were obtained from Merck (Darmstadt, Germany). Analytical grade ethanol was provided by Damao chemical reagent factory (Tianjin, China). Ultrapure water was prepared using a Millipore purified-water system (Brussels, Belgium).

### Preparation and chemical profiling of herbal extracts

RB, SR, and AR were obtained from Anguo County (Hebei, China), Shaanxi Genuine Chinese Herbal Medicine Planting Co., LTD (Shaanxi, China), and Hunyuan County (Shanxi, China), and authenticated as the crude drug of *Rheum palmatum* L., *Scutellaria baicalensis* Georgi., and *Astragalus membranaceus* (Fisch.) Bunge var. *mongholicus* (Bunge) Hsiao, respectively, by Prof. Qingwen Zhang from University of Macau (Macao, China). Fresh GS was collected from Jilin Province of China and authenticated to be the root of *Panax ginseng* (Asian ginseng) by Prof. Songlin Li from Nanjing University of Chinese Medicine (Nanjing, China). The voucher specimen of the four herbs were stored at the herbarium drying cabinet (Eureka, AD-201, Dry Tech Corporation, China) located at our Institute with temperature and humidity controlled (25 ± 5 °C, 50 ± 5% RH). The extracts of SR, AR, GS and RB were prepared as described in Additional file 1. The main components in the four extracts were determined using either HPLC-DAD or HPLC-MS/MS methods and the results were summarized in Tables S1-S4 in Additional file 1.

### In vivo study

Male Sprague–Dawley rats (270–330 g, 8 weeks) were provided by the Experimental Animal Facility of University of Macau (Macao, China) and housed in a temperature (20 ± 2 °C) and relative humidity (45 ± 5%) controlled room with a 12/12 h light/dark cycle under specific pathogen-free conditions. Rats were transferred to the metabolic cages and got acclimatized to the environment for 3 days before starting the experiment. The care and treatment of the rats were in accordance with a protocol (Ref. no.: UMAEC-2015-09) approved by the Animal Ethics Committee, University of Macau.

Thirty rats were divided randomly into 6 groups and received drinking water alone (normal (NOR) group) or 5% DSS in drinking water (UC and herb intervention groups) for consecutive 7 days. In herb intervention groups, rats received a dosage of herbal extract (RB 1.5 g/kg, SR 1.0 g/kg, AR 3 g/kg or GS 1.0 g/kg) suspended in 0.5% carboxymethylcellulose sodium once daily by gavage. The dosage of each herb was determined according to the highest recommended daily dose documented for human in Pharmacopoeia of the People’s Republic of China (2015 version) and the body surface area-based human to animal dosage conversion (conversion factor 6.3). Body weight, rectal bleeding, stool consistency and texture were recorded daily. Feces and urine were freshly collected on days 0, 3, 5, and 7. Blood samples were withdrawn from the orbital sinus on day 7. The dissected colon was cut longitudinally and the contents of the colon were rinsed with phosphate buffer saline (pH 7.4). Then, the length and the weight of the colon were measured to obtain the length/weight ratio as an indicator of colitis severity. Then, colon segments of 1 cm and 0.5 cm from the distal colon were dissected sequentially for myeloperoxidase (MPO) assay and histological assessment, respectively. The remaining colon tissue was reserved for the determination of mRNA levels of IL-4, IL-10, IL-6, IL-1β, TNF-α, TGF-β, monocyte chemoattractant protein-1 (MCP-1), inducible nitric oxide synthase (iNOS), cyclooxygenase-2 (COX-2), and intercellular adhesion molecule-1 (iCAM-1).

### Assessment of colitis

The daily disease activity index (DAI) of each rat was graded based on the body weight loss, rectal bleeding and stool consistency [32]. Colonic MPO assay was carried out as described previously [32] and one unit of MPO activity was defined as the amount of enzyme degrading 1 nM H_2_O_2_ per min at 25 °C. Blood samples were kept at 37 °C for 60 min followed by centrifugation (1000×*g*, 10 min) to obtain serum for determination of cytokines using rat ELISA kits following the manufacturer’s protocol. The colon segment (0.5 cm) was fixed in a 4% paraformaldehyde solution and embedded in paraffin. The 4-µm sections were obtained and stained with hematoxylin-eosin for histological assessment using Olympus CX21 microscope and an Olympus SC100 camera.

### Real-time polymerase chain reaction (RT-PCR) assays of colonic cytokines

The isolation of mRNA, preparation of cDNA, and amplification of target genes by RT-PCR were carried out as previously described [32]. Briefly, total mRNA was extracted from the colon specimens using Trizol reagent and an aliquot (500 ng) was reversely transcribed into first-strand cDNA. Then RT-PCR was performed with the target gene-specific primers (Additional file 1, Table S5) on a ViiA7 QPCR instrument (Thermo Fisher, USA), according to the manufacturer’s protocol. Relative gene expression was calculated using the 2^−∆∆Ct^ method with β-actin as the internal control gene.

### Targeted urinary metabolomics

An aliquot (20 µL) of urine sample was deproteinized with 100 µL of methanol, vortexed for 30 s, and centrifuged (21,500×*g*, 15 min) at 4 °C [4]. The supernatant (5 µL) was separated with an Agilent ZORBAX SB-C18 (100 mm × 2.1 mm, 1.8 μm) column using an Agilent 1200 HPLC system (Agilent, USA) eluted with 0.1% formic acid in H_2_O (A) and 0.1% formic acid in acetonitrile (B) at 200 µL/min under the following gradient program: 0–4 min, 95% A; 8 min, 50% A; 16 min, 0% A; 21 min, 0% A; 23 min, 95% A; 30 min, 95% A. Targeted urinary metabolomics analysis was performed on an 4000 QTrap mass spectrometer (ABSciex, USA) employing scheduled multiple reaction monitoring available in our homemade database which included 410 transitions covering 235 metabolites [33]. Following the acquisition, data were extracted by peak extracting and aligning using the MarkerView software 1.2 (AB Sciex, USA).

### 16S rDNA sequencing analysis of fecal microbiota

Total bacterial DNA was extracted from fecal samples using the QIAamp DNA Stool Mini Kit (QIAGEN, CA). PCR was performed using primers 338F (5′-ACTCCTACGGGAGGCAGCA-3′) and 806R (5′-GGACTACHVGGGTWTCTAAT-3′) targeting the V3–V4 variable region of the 16S rDNA. The following PCR conditions were used: initial denaturation at 98 °C for 30s, followed by 25 cycles consisting of denaturation (98 °C for 15 s), annealing (50 °C for 30s), and extension (72 °C for 30 s) and a final extension step at 72 °C for 5 min.

The sequence of the PCR amplicons was determined on the Illumina MiSeq platform (San Diego, CA). The quality of original sequence data in the FASTQ format was screened one by one using a 10 bp sliding window with 1 bp-length steps, and trimmed at the first window with an average quality value lower than Q20 (i.e., the average base sequencing accuracy ≥ 99%). Subsequently, the paired-end sequence was joined into single-end reads using FLASH (version 1.2.7, http://ccb.jhu.edu/software/FLASH/). Further, sequences that exhibited one of the following characteristics were discarded in the Quantitative Insights into Microbial Ecology (QIIME) software (v1.8.0, http://qiime.org/): sequence length ≤ 150 bp, ambiguous base, mismatched bases, consecutive identical bases ≥ 8. Chimeric sequences were also removed by USEARCH (v5.2.236, http://www.drive5.com/usearch/). Operational taxonomic units (OTUs) were assigned using sequence alignment tool UCLUST. Sequences with > 97% similarity were binned into the same OTU with the most abundant sequence in each OTU selected as the representative sequence. Taxonomic assignment of representative sequences was performed by RDP Classifier (https://sourceforge.net/projects/rdp-classifier/) using the RDP database. All raw sequencing data were available in the NCBI Sequence Read Archive under BioProject number PRJNA851212.

### Data analysis

The multivariate analysis of omics was performed as described in our previous study with minor modifications [4]. Principal component analysis (PCA) and partial least squares discriminant analysis (PLS-DA), hierarchical analysis, and parametric one-way analysis of variance (ANOVA) of microbiome and metabolome data were performed using MetaboAnalyst 4.0 (https://www.metaboanalyst.ca). Briefly, raw data of microbiome and metabolome were subjected to data integrity checking by removing features with at least 50% missing values. The remaining missing values were replaced with a small value (the half of the minimum positive values in the original data). Then, the data were filtered based on the interquartile range to remove baseline noises and normalized by total intensity followed by Pareto scaling to obtain normally distributed variables. ANOVA was carried out to identify distinct metabolites and bacteria with significant differences (*p* < 0.05). All correlation analyses in this study were examined by Spearman correlation test implanted in R package *corrplot* with correlation coefficient > 0.5 and *p* < 0.05 marked as significantly relevant. Heatmap plots were obtained using R package *pheatmap*, and the association network of the distinct gut bacteria, differential metabolites and UC indices was constructed in Cytoscape 3.9.1 software. All other plots were obtained using GraphPad Prism 7.0 software. Unpaired Student’s *t*-test was conducted to determine the differences between two groups and ANOVA was used for comparison among multiple groups. Data were expressed as means ± standard deviation (SD). *P* values < 0.05 were considered statistically significant.

## Results

### The tonic herbs and SR alleviated, while RB aggravated, most symptoms of DSS-induced colitis in rats

**Fig. 1:**
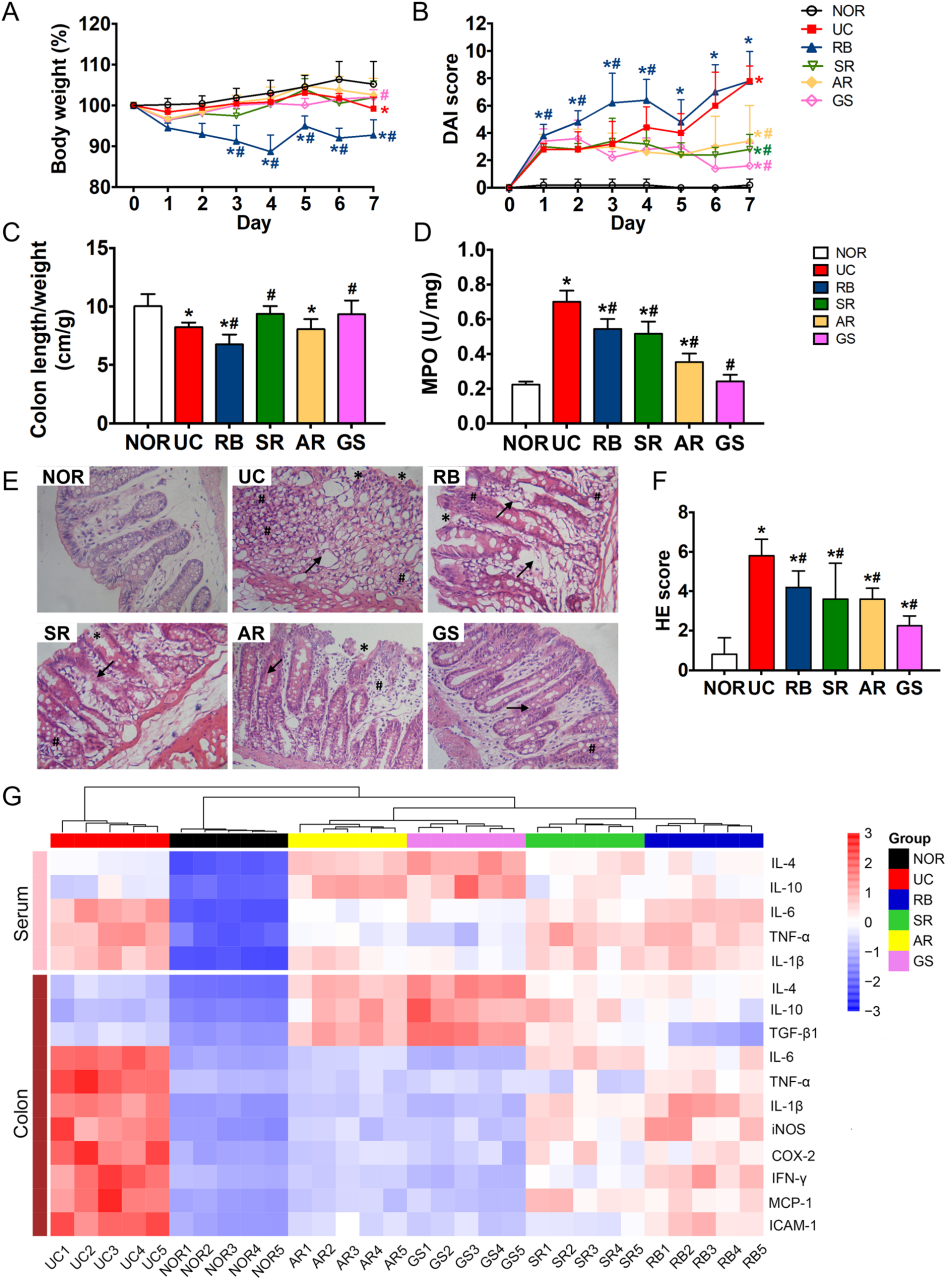
Therapeutical evaluation of four different medicinal herbs on DSS-induced acute colitis rat model. Dynamic changes of **A** body weight and **B** DAI; **C** Rat colon length vs. weight ratio; **D** MPO activity in colon; **E** HE-stained sections of the distal colon, photographs were taken under ×200 magnification. *: mucosa tissue damage; #: inflammatory cells infiltration; →: reduction of goblet cells. **F** HE scores. Data were expressed as mean ± SD. The significance of differences was determined using one-way ANOVA. *p < 0.05 vs. NOR; ^#^*p* < 0.05 vs. UC. *DSS* dextran sulfate sodium, *DAI* disease activity index, *MPO* myeloperoxidase; *HE* Hematoxylin and Eosin

Compared with the NOR group, rats treated with DSS alone exhibited severe diarrhea and rectal bleeding with no weight gain, leading to high DAI during 7 days (Fig. 1A, B). DSS induction also caused remarkably decreased colon length/weight ratio and elevated colonic MPO activity (Fig. 1C, D). Both tonic herbs (GS, AR) and SR slightly increased body weight, alleviated the increases of DAI and MPO, and prevented the decrease of colon length/weight ratio induced by DSS (Fig. 1A–D), while DSS-induced symptoms were aggravated by RB as evidenced by higher DAI mainly attributed to more severe body weight loss, and lower colon length/weight ratio than the UC group. Very interestingly, colonic MPO activity, a sensitive indicator of the influx of inflammatory cells into tissue [34], was also significantly decreased in RB-treated rats.

DSS induction caused the loss of intestinal crypts and goblet cells, severe tissue damage of the epithelial layer, and inflammatory cell infiltration in colon tissues (Fig. 1E, F). The overall tissue damages were attenuated in all herb-treated rats.

Moreover, DSS insult resulted in elevated production of pro-inflammatory (TNF-α, IL-1β, and IL-6) cytokines in serum (Fig. 1G). The levels of anti-inflammatory (IL-4 and IL-10) cytokines were also increased but to less extents. All herbal treatments reversed the increases of the pro-inflammatory cytokines, but further potentiated the elevation of the anti-inflammatory cytokines (Fig. 1G). Similar changes at mRNA levels of these pro- and anti-inflammatory cytokines were observed in the colon tissues. It’s interesting to find that, both tonic herbs also further enhanced the mRNA levels of TGF-β1 which is a multifunctional set of peptides controlling the immune system, while RB reversed its slight increase induced by DSS. SR can suppress the colonic TNF-α elevation to the normal level as efficiently as the tonic herbs (Fig. 1G). Meanwhile, both tonic herbs also mitigated the elevation of the mRNA levels of colonic IFN-γ, MCP-1, iNOS, COX-2, and iCAM-1 induced by DSS.

In general, the two tonic herbs were more effective than the detoxifying SR and/or RB in alleviating clinical colitis symptoms and colonic damages and modulating the aforementioned immune imbalance.

### Herbal interventions elicited faster and greater changes in gut microbial structure than urinary metabolic profile

**Fig. 2:**
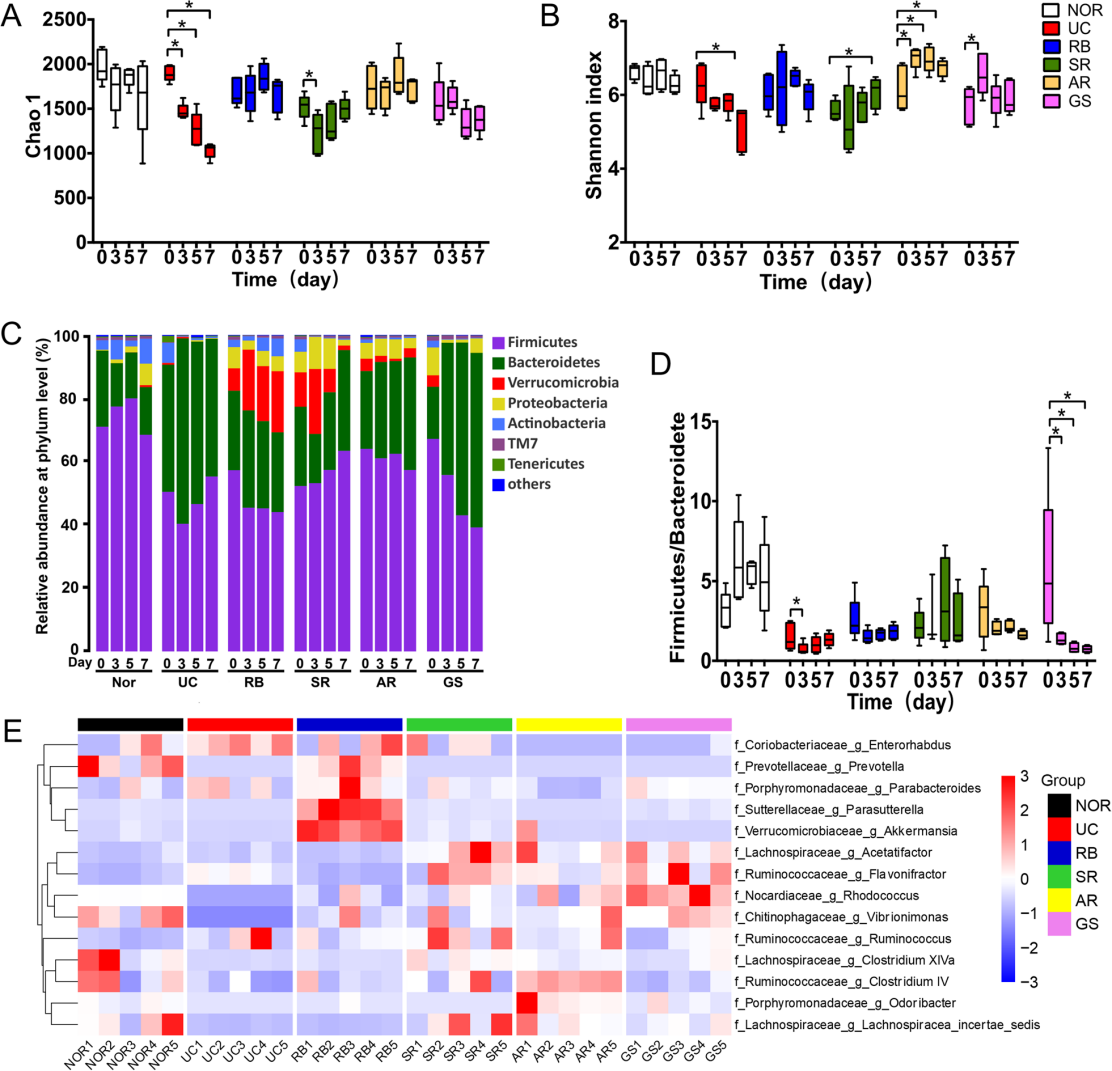
Effect of four different medicinal herbs on the gut microbial structure of DSS-induced acute colitis rat model. The microbial community **A** richness estimator Chao 1 and **B** diversity estimator Shannon index; **C** bar chart of the bacterial community composition at phylum level, **D** the *Firmicutes* to *Bacteroidetes* ratio, and **E** the heatmap of distinct bacteria regulated by UC pathology and tested herbs. Data were expressed as mean ± SD. Significance of differences was determined using one-way ANOVA. *p < 0.05 vs. NOR; ^#^*p* < 0.05 vs. UC.

Compared with the NOR group, the richness and diversity of the microbial community, as indicated by the estimators Chao1 and Shannon, respectively, were gradually reduced in the UC group (Fig. 2A, B), which were altered by herbs of both types in different manners. Specifically, RB abolished DSS-induced changes in both measurements. SR treatment mitigated the decrease in the richness which was finally restored to the original level, and even ended with significantly higher diversity at the end of the treatment. Similar to SR, AR prevented the decrease of the richness and significantly enhanced the diversity. Interestingly, although GS showed a more potent immune-regulatory effect and the strongest alleviation effect on DSS-induced colitis, it only elevated the diversity at the early stage of DSS insult (day 3) while slightly decreased Chao 1.

The alterations in the gut microbial compositions were further compared at phylum level. More than 99% of the total bacteria were assigned into seven dominant phyla (Fig. 2C), namely *Firmicutes*, *Bacteroidetes*, *Verrucomicrobia*, *Proteobacteria*, *Actinobacteria*, *Candidatus Saccharibacteria (TM7)*, and *Tenericutes*. An increase of *Bacteroidetes* was observed in the UC group, leading to a decreased *Firmicutes/Bacteroidetes* (F/B) ratio at the early stage (day 3, Fig. 2D). RB treatment resulted in a decrease of *Firmicutes*, corresponding to lowered F/B ratios, while SR generally increased the abundance of both *Firmicutes* and *Bacteroidetes* and as a consequence maintained the F/B ratio throughout the experimental period. In contrast, GS intervention resulted in markedly decreases in *Firmicutes* and increases in *Bacteriodetes*, causing significantly decreased F/B ratios. AR altered the two main phyla in the same manner as GS but to a much lesser extent (Fig. 2C, D). The abundance of *Verrucomicrobia* in the two groups receiving the detoxifying herbs was much higher than those in the tonic herb groups, which was eliminated by GS treatment. All the four herbs can maintain relatively higher abundance of *Proteobacteria* which was minor in the UC group, while only RB recovered the abundance of *Actinobacteria* which was significantly reduced by DSS insult.

At genus level (Fig. 2E), 14 genera showed differential relative abundance among six experimental groups. Specifically, DSS insult resulted in the increases of *Enterorhabdus* and *Flavonifractor* as well as decreases of six genera (*Prevotella*, *Rhodococcus*, *Vibrionimonas*, *Clostridium XIVa*, *Clostridium IV*, and *Lachnospiracea incertae sedis*). All four herbs mitigated DSS-induced decreases of *Rhodococcus* and *Vibrionimonas* with the tonic herbs more potent than the detoxifying herbs. Both tonic herbs displayed similar effects on five genera with GS more effective, specifically, suppressed DSS-induced elevation of *Enterorhabdus*, potentiated the increase of *Flavonifractor*, while reversed the suppression of *Rhodococcus* and *Vibrionimonas* and enhanced the levels of *Acetatifactor* which was unaffected by DSS insult. Additionally, AR potentiated the levels of three extra genera (*Clostridium IV*, *Odoribacter*, and *Lachnospiracea incertae sedis*) which were unaltered by GS treatment. The impact of SR on the gut microbial structure is largely similar to the tonic herbs, except for potentiating DSS-induced elevation of *Ruminococcus* and restoring the reduced *Lachnospiracea incertae sedis*. Interestingly, in addition to the reversal of the decreases of *Prevotella*, *Rhodococcus*, and *Vibrionimonas* and the increase of *Flavonifractor* triggered by DSS, RB intervention also elicited distinct microbial alterations, resulting in marked increases of *Parasutterella* and *Akkermansia* which were unaltered in DSS-induced colitis and by other herb treatments. Further hierarchical clustering analysis of gut microbiota structure on day 5 and day 7 revealed a clear separation between the Normal and UC group (Additional file 1: Fig. S1). Agreeing with the genus-level changes, SR and AR were always clustered together, while GS gradually moved away from SR and AR and is clustered into a separate group on day 7. Interestingly, RB displays a distinct pattern in gut microbial structure, making it well separated from all other treatments on both day 5 and day 7.

**Fig. 3:**
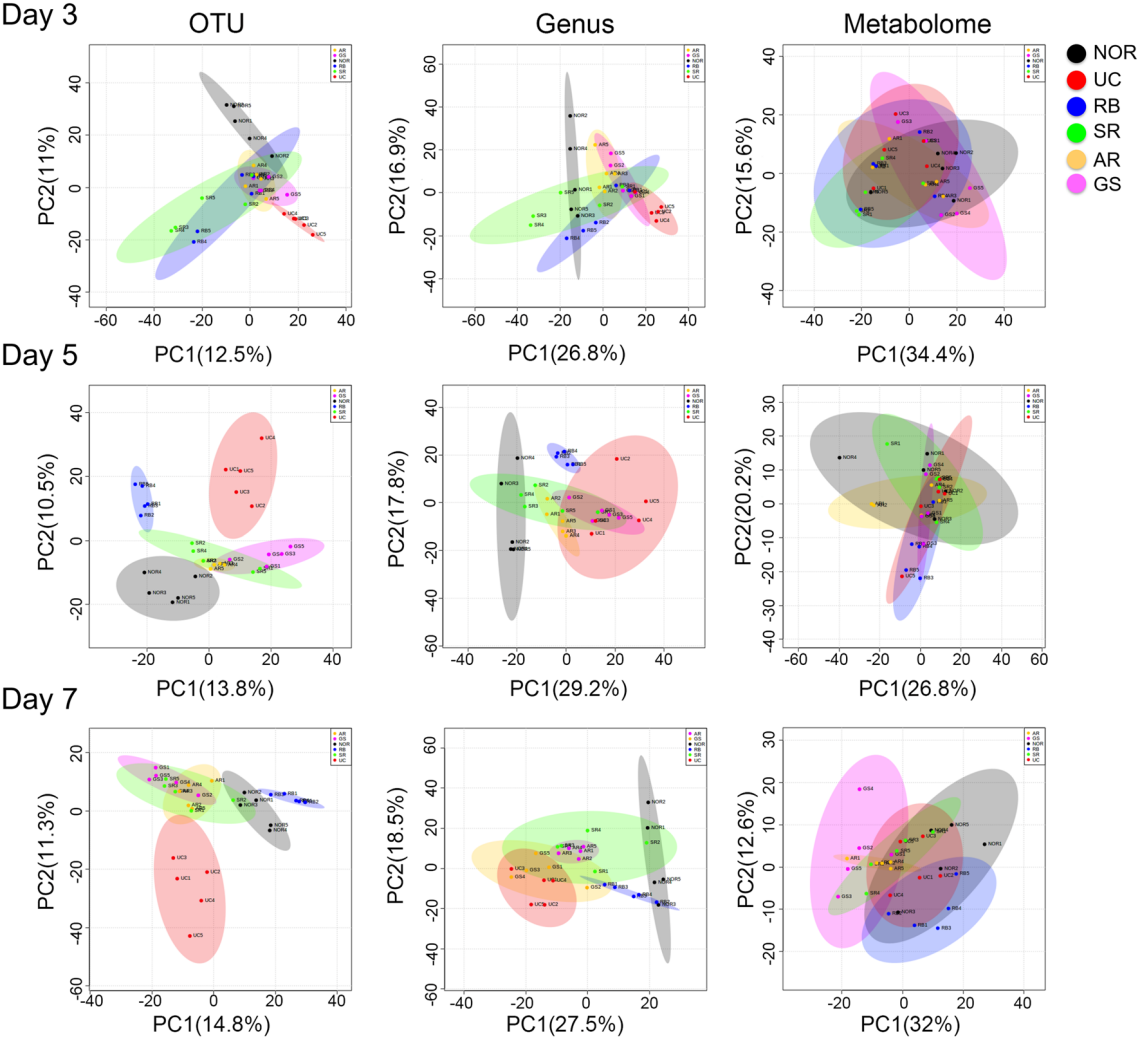
Principal component analysis (PCA) of bacterial OTUs, genus and host urinary metabolome on days 3, 5, and 7. Groups in the plot are shown with 95% confidence ellipses

Further PCA analysis revealed that the microbial structure at both OTU and genus levels of the UC group markedly shifted away with changes at OTU level essentially separated from the normal status throughout day 3 to day 7 (Fig. 3). In presence of herb intervention, the trajectories of microbial changes drifted away from the colitis group to different extents and in different directions as well. Specifically, the bacterial OTU profile of the RB group on days 5 and 7 was well separated from those of the other three herbal groups in the PC1 and positioned on opposite sides of the normal group (Fig. 3). Although the other three herb-treated groups, including the tonic GS and AR and the detoxifying SR, clustered together and separated from the UC group, they also well separated from the normal group (Fig. 3). The gut microbial structures at genus level were generally less distinguishable than that at OTU level. Only the RB group was still well separated from all other groups, agreeing with the clustering analysis (Additional file 1: Fig. S2). GS moved away from the UC group on day 7, while SR and AR are still partially overlaid with the UC group.

The PCA plot of the urinary metabolome showed high intra-group variations and could not separate different groups from each other (Fig. 3). Supervised PLS-DA plots (Additional file 1: Fig. S2) showed similar patterns to those of the PCA analysis (Fig. 3). The microbiome at OTU level was generally more distinguishable than that at the genus level. The PLS-DA plots revealed high intra-group variations in the urinary metabolome and could not separate different groups from each other on days 3 and 5. Although the colitis group was still slightly overlaid with NOR on day 7, both groups receiving the tonic herbs shifted away from the colitis group and partially merged with NOR.

### Perturbations of the gut microbiome and host urinary metabolome displayed increasing associations

**Fig. 4:**
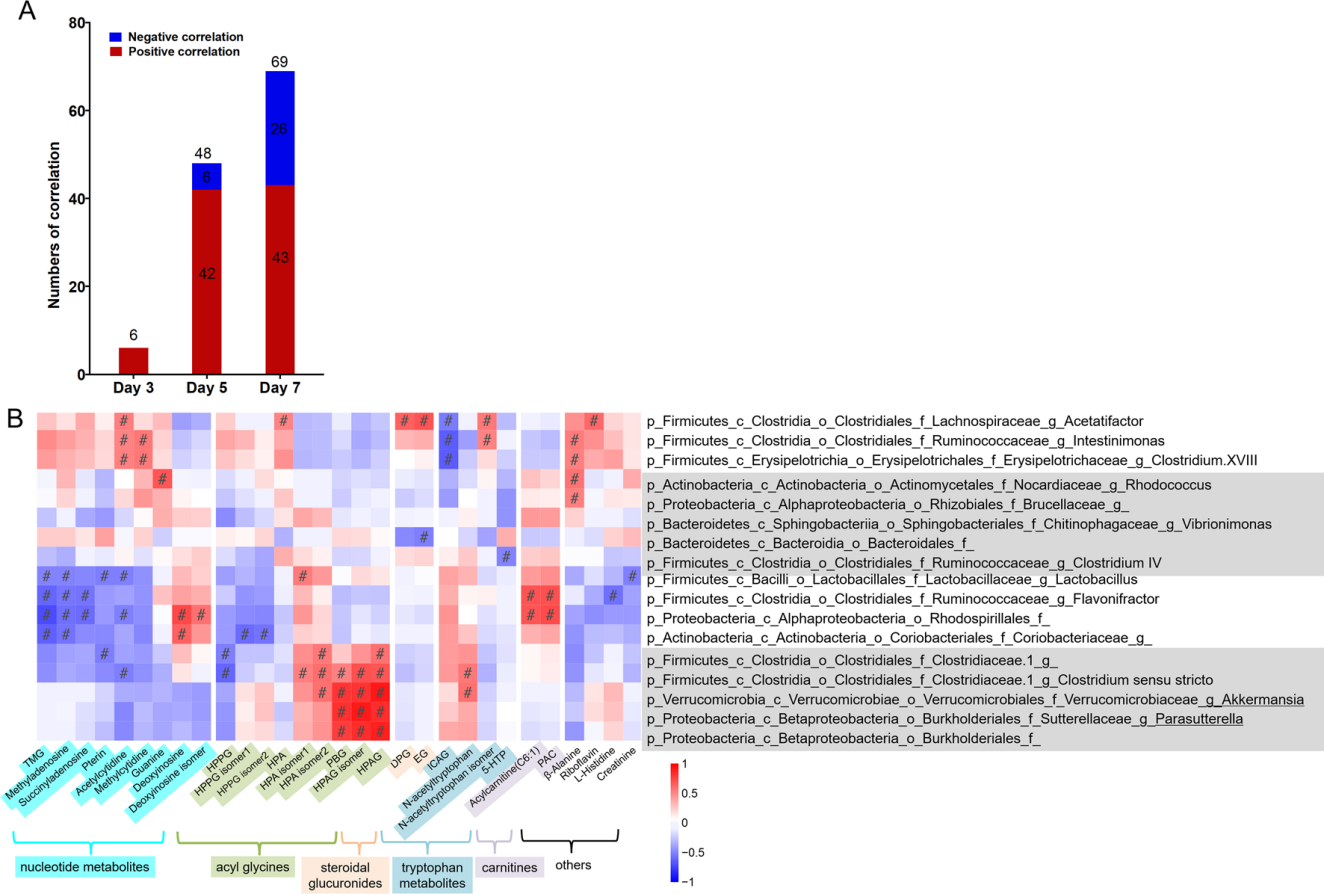
Gut microbiome and host urinary metabolome displayed increasing associations. **A** The number of significant correlations between distinct genera and metabolites on days 3, 5 and 7. **B** heatmap of significant correlations between differential genera and metabolites on day 7. #: linear correlation coefficient R > 0.5 and *p* < 0.05. *TMG* trimethylguanosine, *HPPG* hydroxyphenylpropionylglycine, *HPA* hydroxyhippuric acid, *PBG *phenylbutyrylglycine, *HPAG* hydroxyphenylacetylglycine, *DPG* dehydropregnenolone glucuronide, *EG* estriol-glucuronide, *ICAG* 3-indole carboxylic acid glucuronide, *5-HTP* 5-hydroxytryptophan, *PAC* phenylacetyl-carnitine

To decipher whether there was an overall association between the perturbed gut microbiome and host metabolome during the development of colitis, linear correlation analysis was performed between PC1s of PLS-DA plots of bacterial OTUs and those of host urinary metabolome on days 3, 5, and 7 (Additional file 1: Fig. S3). The linear coefficient R was 0.439, 0.508, and 0.661 on days 3, 5, and 7, respectively, indicating increasing associations between host urinary metabolome and bacterial OTUs. Pairwise Spearman correlation analysis further revealed that the numbers of significant correlations (*p* < 0.05) between differential genera and metabolites increased dramatically (6, 48 and 69 on days 3, 5 and 7, respectively) (Fig. 4A). The correlations were all positive on day 3, while the numbers of negative correlations increased dramatically from 6 on day 5 to 26 on day 7 (12.5% and 37.7% of total correlations, respectively).

On day 7, 30 distinct metabolites (mainly including nine acyl glycines, nine nucleotide metabolites, four tryptophan metabolites, two steroidal glucuronides, and two acylcarnitines) displayed significant association with 17 differential bacterial genera (including eight from *Firmicutes*, four from *Proteobacteria*, two from *Actinobacteria*, two from *Bacteroidetes* and one from *Verrucomicrobia*) with 69 correlations (26 negative and 43 positive) reaching statistical significance (Fig. 4B). Generally, the associations among the acyl glycines (hydroxyhippuric acid (HPA) isomers, phenylbutyrylglycine (PBG), hydroxyphenylacetylglycine (HPAG) and HPAG isomer) and five bacteria at different phylogenetic classification levels, namely the genus *Clostridium sensu stricto* and the family *Clostridiaceae* of phylum *Firmicutes*, the genus *Parasutterella* and the order *Burkholderales* of phylum *Proteobacteria* and the genus *Akkermansia* (*Akkermansia muciniphila*) of the phylum *Verrucomicrobia*, make the biggest contribution to the positive correlation (total 17 positive correlations). The three genera *Acetatifactor*, *Intestinimonas* and *Clostridium XVIII* under phylum *Firmicutes* made the second contribution with 13 positive correlations among metabolites spanning over all different chemical groups, namely the nucleotides (acetylcytidine and methylcytidine), acyl glycines (HPA), steroidal glucuronides (dehydropregnenolone glucuronide (DPG), estriol glucuronide (EG)), the tryptophan metabolites (*N*-acetyltryptophan isomer), and others (β-alanine and riboflavin). For the negative correlations, four bacteria, including the two genera *Lactobacillus* and *Flavonifractor* from *Firmicutes*, the family *Coriobacteriaceae* from *Actinobacteria* and the order *Rhodospirillales* from *Proteobacteria*, are the main contributors and established 17 significant correlations with many metabolites in the nucleotide group (trimethylguanosine (TMG), methyladenosine, succinyladenosine, pterin, acetylcytidine), acyl glycines (hydroxyphenylpropionylglycine (HPPG) and two isomers), and others (l-histidine and creatinine). It’s interesting to note that the association analysis revealed the distinct relationships of different groups of bacteria to one specific chemical type, for instances, the three genera *Acetatifactor*, *Intestinimonas* and *Clostridium XVIII* were positively associated to most nucleotide metabolites, while the six bacteria (the three genera *Lactobacillus*, *Flavonifractor*, and *Clostridium* *sensu stricto*, the two families *Clostridiaceae 1* and *Coriobacteriaceae*, the order *Rhodospirillales*) displayed negative correlations. Notably, the same group of bacteria can correlate distinctly (e.g., HPA and its two isomers, *N*-acetyltryptophan and its isomer) or in similar profiles (e.g., the steroidal glucuronides DPG and EG, the acylcarnitines acylcarnitine (C6:1) and phenylacetyl-carnitine (PAC); deoxylnosine and deoxylinosine isomer) with chemical analogs.

Further inspection of the results found that the tonic herbs, especially GS, made a higher contribution to the positive correlations between genus *Rhodococcus* and the two metabolites β-alanine and guanine (Additional file 1: Fig. S4A). RB is the sole contributor to the strong correlations between the acyl glycines HPAG and PBG and the genera *Akkermansia* and *Parasutterella* (Additional file 1: Fig. S4B), while SR is the one accounting for the correlation between the genus *Acetatifactor* and both steroidal glucuronides (Additional file 1: Fig. S4C).

### RB and SR triggered significant alterations in steroidal glucuronides and acylglycines, respectively

**Fig. 5:**
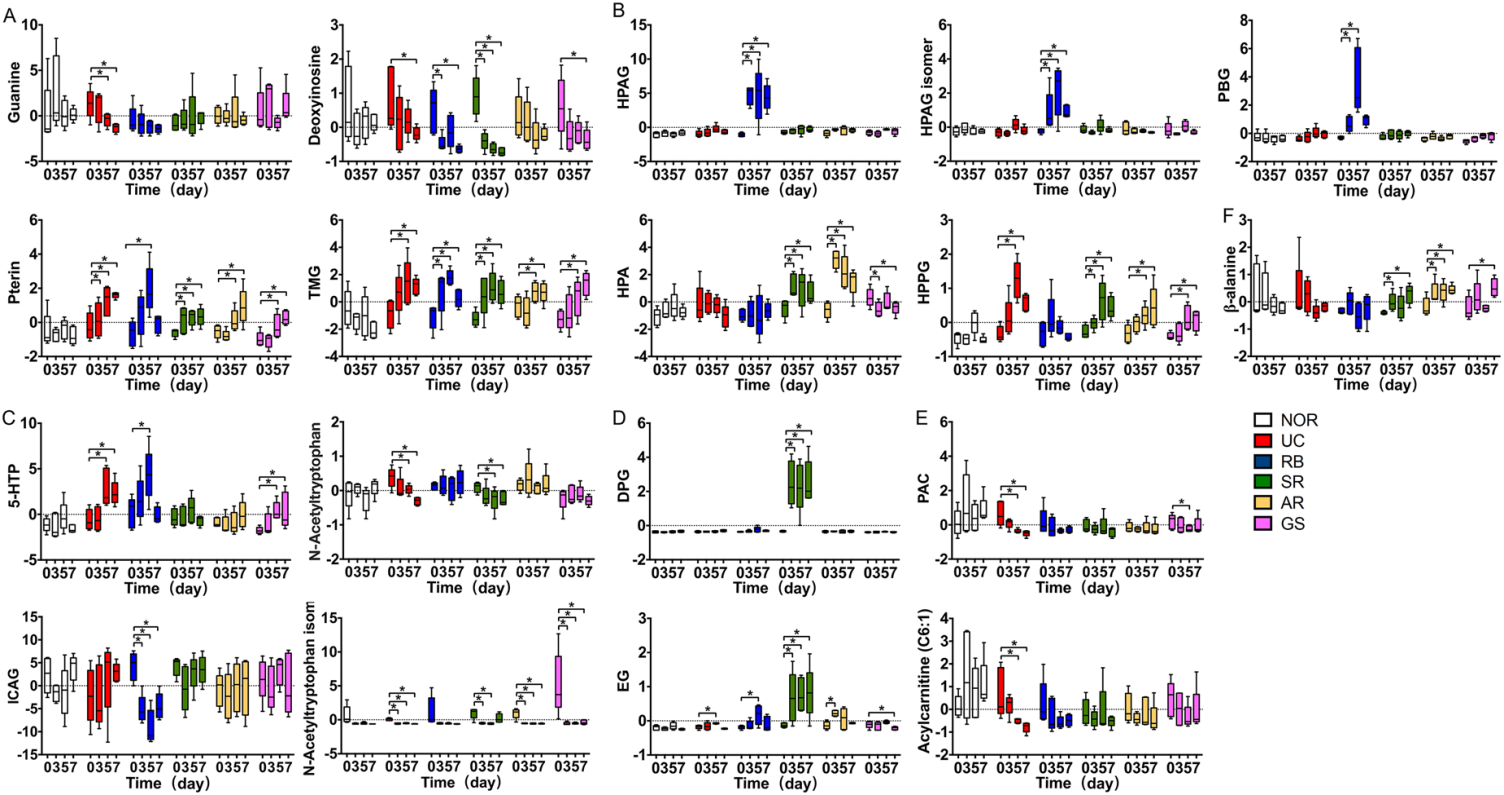
Relative abundance of distinct metabolites. **A** nucleotide metabolites: guanine, deoxyinosine, pterin, trimethylguanosine (TMG); **B** acyl glycines: hydroxyphenylpropionylglycine (HPPG), hydroxyhippuric acid (HPA), hydroxyphenylacetylglycine (HPAG), phenylbutyrylglycine (PBG); **C** tryptophan metabolites: *N*-acetyltryptophan and isomer, hydroxytryptamine (5-HTP), 3-indole carboxylic acid glucuronide (ICAG); **D** steroidal glucuronide conjugates: dehydropregnenolone glucuronide (DPG) and estriol-glucuronide (EG), **E** carnitines: phenylacetyl-carnitine (PAC) and acylcarnitine (C6:1), and **F** β-alanine in different groups. Data were expressed as mean ± SD. *p < 0.05

The differential metabolites induced by DSS and those by each herbal intervention were further analyzed (Fig. 5). DSS insult elicited gradual decreases of guanine, deoxyinosine, *N*-acetyltryptophan and isomer, PAC and acylcarnitine (C6:1) throughout the experimental period. In contrast, pterin, TMG, HPPG, 5-hydroxy-tryptophan (5-HTP), and EG were significantly increased in DSS-induced colitis, peaking at day 5 while maintaining or decreasing on day 7. The effects of the two tonic herbs on the metabolic shifts are largely similar: both could abolish (guanine, *N*-acetyltryptophan) or mitigate (HPPG, 5-HTP, PAC, acylcarnitine (C6:1)) most aforementioned changes. Although the detoxifying herb SR shared some common features with RB, such as potentiating DSS-induced decrease of deoxyinosine, its actions on DSS-induced changes more resemble those of the two tonic herbs. Particularly, both tonic herbs and SR increased the level of alanine, which was unchanged after DSS insult or RB intervention; both AR and SR caused significant increases in HPA, a microbial aromatic acid metabolite, which was not observed in DSS induction. It is interesting to find that the two detoxifying herbs elicited distinct metabolic alterations. Specifically, RB abolished DSS-induced elevation of the acyl glycine HPPG, did not alter the level of HPA which was elevated by SR and AR, but induced significant and unique increases of PBG, HPAG and its isomer. RB also significantly decreased the level of 3-indole carboxylic acid glucuronide (ICAG), a glucuronide of indole pathway of tryptophan metabolism, which was not affected by DSS insult or any other herbal intervention. In contrast, SR treatment resulted in the elevation of EG and DPG, although the level of ICAG was unaltered. The changes of the two steroidal glucuronides showed a high correlation with that of genus *Acetatifactor* with sole contribution by SR (R values 0.916 and 0.977) (Additional file 1: Fig. S4C).

### Crosstalk among gut microbiome-metabolome-UC axis

**Fig. 6:**
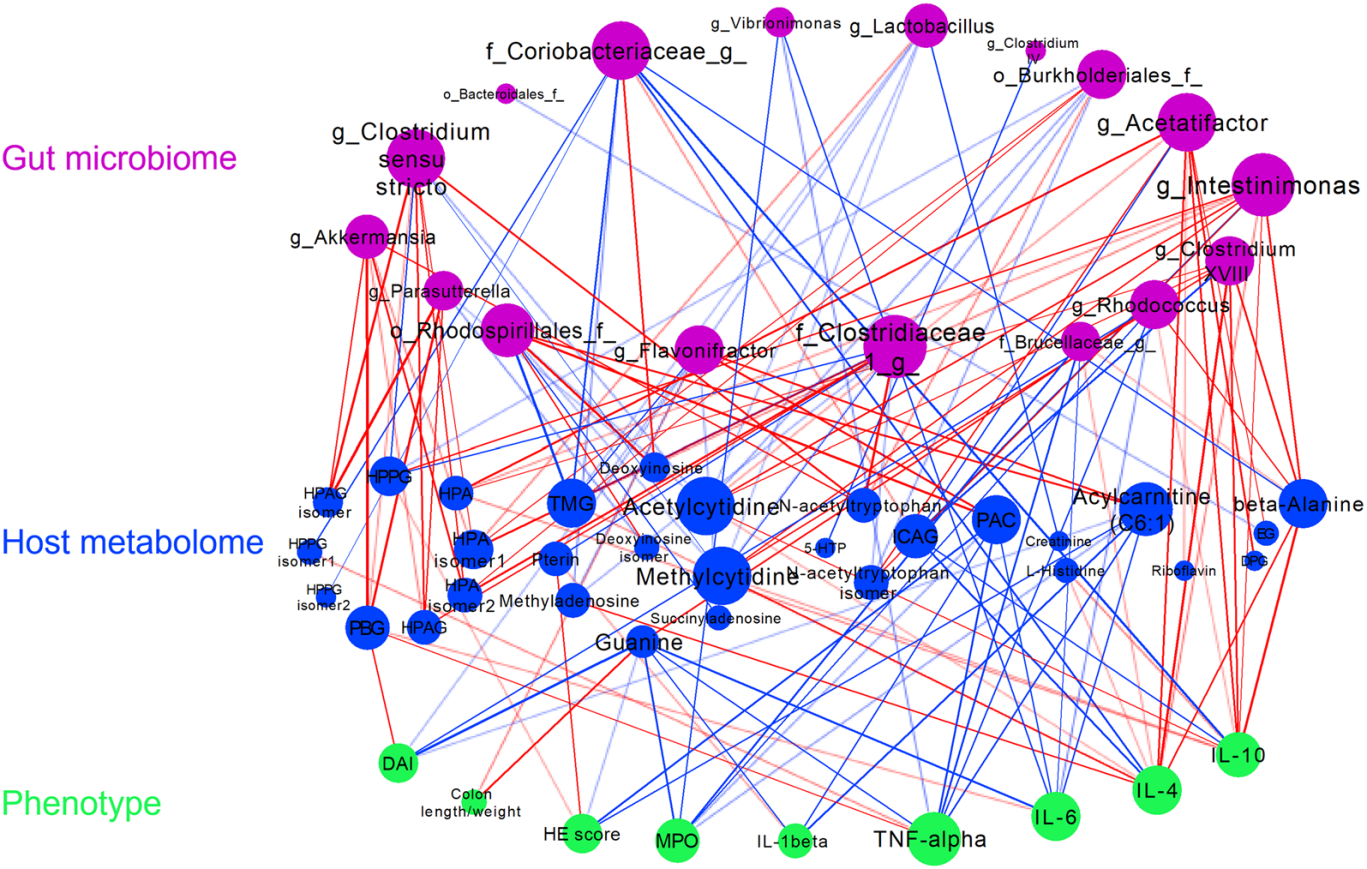
Correlation network among discriminative gut microbial taxa, differential metabolites and UC indices (Spearman coefficient > 0.5 and p < 0.05). The red line represents the positive correlation, and the blue line represents the negative correlation. The stronger the interaction, the thicker and darker a line is. The node size represents degree. The more correlations with other elements, the bigger a node is. *TMG* trimethylguanosine, *HPPG* hydroxyphenylpropionylglycine, *HPA* hydroxyhippuric acid, *PBG* phenylbutyrylglycine, *HPAG* hydroxyphenylacetylglycine, *DPG* dehydropregnenolone glucuronide, *EG* estriol-glucuronide, *ICAG* 3-indole carboxylic acid glucuronide, *5-HTP* 5-hydroxytryptophan, *PAC* phenylacetyl-carnitine, *DAI* disease activity index, *MPO* myeloperoxidase, *HE* Hematoxylin and Eosin

To further assess whether there exist associations among gut microbiome, host metabolome and therapeutical effect of herbs, an interaction network was constructed with data from 3 dimensions: 17 differential gut microbial genera, 30 distinct metabolites and 9 phenotypic measurements (Fig. 6). It is interesting to find that the phenotype data were integrated into the network. In particular, two anti-inflammation cytokines (IL-4 and IL-10) showed similar correlation profiles. They simultaneously showed strong positive correlation with three genera i.e., *Acetatifactor*, *Clostridium*, and *Intestinimonas*, as well as negative correlation with the family *Coriobacteriaceae*. Meanwhile, they also positively correlated with two nucleotides (methyladeosine and acetylcytidine), l-histidine and β-alanine, while negatively with ICAG. Three pro-inflammatory cytokines (IL-1β, TNF-α and IL-6) and MPO displayed most negative associations with metabolites guanine, PAC, acylcarnitine (C6:1), and bacteria including genera *Vibrioniomons* and *Rhodcoccus*, family *Brucellaceae*. Other UC indices, including DAI, HE scores and MPO, negatively related to guanine, while the ratio of colon length to weight showed a positive correlation with guanine.

## Discussion

In TCM practice, reinforcing the vital *qi* with tonic herbs or eliminating the pathogenic factors with detoxifying herbs are two main principles of treatment when compound formulas are prescribed to alleviate gastrointestinal disorders, including UC [10, 11]. The present study first tries to understand the tonic and detoxifying properties of CMs from the viewpoints of the gut microbiome and the associated host metabolome. The results showed that although sharing some common features in DSS-induced metabolic and gut microbial alterations, the tonic herbs and detoxifying herbs alleviated colitis symptoms differently and elicited alternative gut microbial and host metabolic reprogramming. The common and differential effects are by and large in concert with the applications of these herbs in TCM practice. Specifically, the main findings include (1) both tonic herbs were more effective in alleviating DSS-induced acute colitis, while RB exacerbated the colitis clinical symptoms when alleviated inflammation; (2) The metabolic network shifts were largely similar between the two tonic herbs, while the changes elicited by SR more resembles the tonic herbs, rather than RB; (3) Both detoxifying herbs elicited unique microbial and metabolic changes which agree with their different targeting sites in the triple energizer (*Sanjiao*; the upper- and the lower-energizer for SR and RB, respectively) according to the TCM theory. RB-specific increases of acyl glycines highly correlated with the increased abundance of *Akkermansia muciniphila* and the genus *Parasutterella*, while SR-specific elevation of some steroidal glucuronides exhibited a significant positive correlation with the genus *Acetatifactor*.

UC is characterized by immune imbalance. DSS induction resulted in marked increases of both pro-and anti-inflammatory cytokines in serum which is consistent with the previous report [26]. The tonic and detoxifying herbs affected the immune balance differentially (Fig. 1E, F). Both tonic herbs consistently potentiated the production of anti-inflammatory cytokines and suppressed the pro-inflammatory cytokines. SR and RB generally displayed similar but much weaker effects with the exception that RB and SR mitigated DSS-induced colonic TGF-β1 and TNF-α production to normal levels, respectively. These findings indicate that the immunomodulatory effect of tonic herbs is more important for their colitis alleviating benefits. The laxative effects of the anthraquinone ingredients may mask the health benefits at the tested dosage of RB, causing the exacerbated clinical symptoms [35].

The alterations of gut microbial structure elicited by DSS insult as well as herbal interventions occurred faster (obvious changes observed on day 3) and greater than the metabolic shifts, supporting that as the main constituent of the gut barrier, the gut microbial community serves as a more sensitive pathologic/pharmacological indicator than the metabolic biomarkers in the early stage of disease/drug intervention. Altered F/B ratios were commonly observed in diseases such as obesity and IBD, and thus used as an important indicator of disease progression and/or intervention [36]. The increase of phylum *Firmicutes* and the decrease of phylum *Bacteroidetes* resulted in an increased F/B ratio in UC patients [2]. However, the preclinical study reported decreased F/B ratio in DSS-induced acute colitis rats [30]. In this study, fecal samples were collected at multiple time points during the development of acute colitis, which allowed us to characterize the dynamics of the relative abundance of the two phyla. DSS insult resulted in a dual profile (decrease then restore) of changes, indicating that the F/B ratio may differ with disease stage. All four herbs changed the F/B ratio but in different manners. Even though both tonic herbs markedly alleviated colitis symptoms, they did not reverse (AR) or even potentiated (GS) DSS induction-caused decreases of F/B ratio which were achieved mainly through an elevation of *Bacteriodetes* (AR) or an increase of the *Bacteroidetes* as well as a decrease of the *Firmicutes* (GS). Therefore, all tested herbs seem to reinstate the microbial balance differently instead of restoring it to the initial state. Moreover, given that both phyla consist of a large number of bacterial species and each of which may respond differently to DSS insult or herbal intervention, the F/B ratio is not a suitable measurement to assess the impact of disease and interventions on the microbial structure.

The tonic herbs share more common features than the detoxifying herbs in microbiome and metabolome alterations. The colitis alleviating effects of the tonic herbs could be partially ascribed to elevating the relative abundance of some beneficial bacteria such as *Rhodococcus* (Fig. 2E). Most bacteria under the genus *Rhodococcus* are benign and can degrade organic toxins that induce inflammation and metabolic diseases, such as phenols, biphenyls, and their derivatives [37, 38]. Both tonic herbs up-regulated *Flavonifractor* and *Acetatifactor* and suppressed DSS-induced elevation of genus *Enterorhabdus*. *Acetatifactor* is a bacterial genus from the family of *Lachnospiraceae*. Up to now *Acetatifactor muris* is the only known species of this genus [39]. *Flavonifractor* [40] and *Acetatifactor* [39] are both short-chain fatty acid (SCFA) producers. Gut microbiota produces SCFAs through fermenting indigestible dietary components to promote epithelial integrity and exert anti-inflammation activity [41]. The capacity of butyrate restoration and production was associated with sustained remission in UC patients receiving FMT [5]. An increase of *Flavonifractor* by dietary resveratrol could alleviate weaning-associated diarrhea and intestinal inflammation in pig offspring [40]. On the other hand, *Enterorhabdus* strains *E. mucosicola* and *E. caecimuris* have been isolated from a spontaneous colitis mouse model [42]. Both tonic herbs can decrease the relative abundance of *Enterorhabdu*s. AR treatment also restored *Clostridium IV* which was decreased by DSS induction (Fig. 2E). The decrease of *Clostridium IV* was reported in UC patients [5]. Inoculation of a mix of *Clostridium* strains during the early life of conventionally reared mice resulted in resistance to colitis and systemic immunoglobulin E responses in adult mice [43]. As such, *Clostridium* could serve as an important predictor for UC remission by herbal interventions in our study. AR also enhanced the abundance of *Odoribacter. Odoribacter* was enriched in healthy people and positively correlated with improved metabolic features in patients with metabolic syndrome [44].

A growing body of evidence indicated an essential role of gut microbiota in maintaining the host metabolic homeostasis via multi-dimensional host-microbe metabolic interactions [5, 15]. Although this study revealed insignificant global metabolic shifts in DSS-induced acute colitis, the dramatically increased microbiome-metabolome correlations indicate an ever-increasing contribution of gut microbiota to the global host metabolic profile during colitis development (Fig. 4).

GS intervention caused significant enrichment of *Rhodococcus* and made a larger contribution to the positive correlations between *Rhodococcus* and guanine and β-alanine. High guanine–cytosine content is characteristics of *Rhodococcus* species which provides DNA stability in varying conditions [45]. Guanine and derivatives or analogs can activate immune cells via toll-like receptor 7 (TLR7) and stimulate both humoral and cellular immune responses [46]. Meanwhile, guanine negatively correlated with inflammation-related UC indices including HE score, MPO, IL-1β, TNF-α and IL-6 and positively correlated the ratio of colon length to weight, supporting the immunomodulatory effect of guanine in alleviating UC (Fig. 6). β-Alanine exerts immunoregulatory effects by activating both T and B cells [47] and was positively related to anti-inflammation cytokines IL-4 and IL-10 (Fig. 6). GS abolished the DSS-induced decreased tendency of guanine and β-alanine (Fig. 5A, F), which can explain the strong immunoregulatory effect of the herb.

In the serotonin pathway, tryptophan was uptaken by enterochromaffin cells, where it was converted to 5-HTP by tryptophan hydroxylase followed by decarboxylated to 5-hydroxytryptamine (5-HT) by decarboxylase [48]. A high level of 5-HT can result in increased colitogenic microbiota [49]. SR and AR significantly suppressed the DSS-induced increase of 5-HTP which displayed a significant negative correlation with the genus *Clostridium IV*. Given that *Clostridium IV* was significantly enriched by AR intervention, this bacterium and the tryptophan pathway should be the main contributor to the therapeutic outcomes of AR.

RB-specific enrichment of the genera *Akkermansia* and *Parasutterella* correlates well with the elevation of the acyl glycines PBG, HPAG and its isomer. Increased acylglycine excretion was directly related to the intra-mitochondrial accumulation of the corresponding acyl-CoA esters [50]. Therefore, the elevation of these acetylglycines in RB group signifies the mitochondrial fatty acid β-oxidation disorder, which should contribute to aggravated colitis symptoms by RB treatment. UC indices including DAI, TNF-α and IL-6 were positively correlated with PBG. As the only member of the genus *Akkermansia*, *Akkermansia muciniphila* is an SCFA producer and uses mucin as its sole energy source in the gut [51]. It also reversed the high fat-induced pro-inflammatory cytokine IL-6 and visceral adipose tissue inflammation through Treg induction [52]. The abundance of *Parasutterella* showed a positive correlation with the inflammatory cells in subcutaneous tissue during irritable bowel syndrome development and progression in patients [53]. RB intervention also resulted in the enrichment of *Prevotella*. Intestinal *Prevotella* species (*Prevotella intestinalis* nov. sp.) colonization resulted in reduced SCFA production by gut microbiota and reduced IL-18 level, consequently enhancing host susceptibility to mucosal inflammation [54]. Taken together, the enrichment of *Akkermansia* may partially explain the anti-inflammatory effect of RB, while the elevation of *Prevotella* and *Parasutterella* may contribute to aggravated colitis symptoms by RB.

SR treatment resulted in significant increases of two steroidal glucuronides EG and DPG. The levels of these endogenous glucuronides are the result of coordinated efforts of host uridine 5′-diphospho-glucuronosyltransferases (UGTs)-mediated glucuronidation and gut microbial β-glucuronidases (GUSs) catalyzed deglucuronidation. The glucuronic acid conjugation is considered as a common “detoxifying” mechanism of numerous endogenous/exogenous compounds through facilitating the excretion of the more water-soluble glucuronidated metabolites into urine [24]. Gut microbial GUSs-catalyzed deglucuronidation of glucuronidated metabolites in gut lumen will facilitate their reabsorption, which resulted in enhanced local and/or systemic exposure. Some main components of SR, such as baicalin and baicalein, have been reported to be the inhibitors of gut microbial GUSs [55]. Moreover, SR is the sole contributor to the significant positive correlations between the two steroidal glucuronides and the genus *Acetatifactor* (R > 0.9, Additional file 1: Fig. S4). These findings imply an important role of *Acetatifactor* in the colitis alleviating effect of SR and also highlight the potential of the host UGTs-microbial GUSs axis as a strategy for colitis intervention. A further study is warranted to investigate the role of host UGTs-gut microbial GUSs axis in metabolic homeostasis of steroidal hormones and colitis initiation and development.

## Conclusion

This study unraveled the gut microbial alterations and the associated host metabolic shifts in response to the interventions by four individual herbs characterized by tonic or detoxifying properties. Generally, both tonic and detoxifying natures could promote UC remission, although via alternative microbial and host metabolic reprogramming. The two tonic herbs showed higher efficacies than the two detoxifying herbs in alleviating acute colitis clinical symptoms and shared more common profiles in gut microbial alterations and metabolic shifts. Each detoxifying herb elicited unique microbial changes and associated metabolic signatures, which could be ascribed to different chemical types of main bioactive herbal components, distinct targeting sites and severe laxative effects of anthraquinones at the tested dosage of RB. Although the study was carried out with only two herbs from each category at only one dosage of each converted from the highest dosage recommended for human in an acute colitis animal model, these findings offer some specific microbial and metabolic signatures for modern scientific understanding of the tonic and detoxifying properties of CMs in TCM theory which might involve different microbiome and metabolome reprogramming via diverse as well as distinct mechanisms. Further studies on chronic colitis are also warranted for a comprehensive understanding of the tonic and detoxifying properties of CMs and their differential roles at different disease stages. Validation of the roles of related microbe and metabolites in colitis pathology will also offer new strategies/targets for colitis intervention.

## Supplementary Information

**Additional file 1.** Additional methods. **Figure S1.** Hierarchical analysis of gut microbial composition on day 5 and day 7. **Figure S2.** PLS-DA of bacterial OTUs, genus, and host urinary metabolome on days 3, 5, 7. **Figure S3.** Correlation analysis between PC1s of PLSDA plot of bacterial OTUs and urinary metabolome on day 3, 5 and 7. **Figure S4.** Correlation analysis between **A** genus Rhodococcus and the metabolites β-alanine, guanine and creatinine; **B** genus Akkermansia and Parasutterella and the metabolites hydroxyphenylacetylglycine (HPAG) and phenylbutyrylglycine (PBG); **C** genus Acetatifactor and estriol-glucuronide (EG). **Table S1.** MRM ion pairs and calibration curves for quantitative analysis of main anthraquinones and the contents in RB. **Table S2.** Wavelengths and calibration curves for quantitative analysis of main flavonoids and the contents in SR. **Table S3.** MRM ion pairs and calibration curves for quantitative analysis of main components and their contents in Astragali Radix extract. **Table S4.** MRM ion pairs and calibration curves for quantitative analysis of ginsenosides and their contents in ginseng extract.

## Data Availability

The data analyzed during this study can be obtained from the corresponding author on reasonable request.

## Abbreviations

UC: Ulcerative colitis
IBD: Inflammatory bowel disease
FMT: Fecal microbiota transplantation
DSS: Dextran sodium sulfate
CM: Chinese medicine
RB: Rhubarb
AR: Astragali Radix
GS: *Panax ginseng*
SR: Scutellaria Radix
DAI: Disease activity index
MPO: Myeloperoxidase
TNF-α: Tumor necrosis factor α
IL-1β: Interleukin 1β
IL-6: Interleukin 6
IL-4: Interleukin 4
IL-10: Interleukin 10
TGF-β1: Transforming growth factor-1β
MCP-1: Monocyte chemoattractant protein-1
iNOS: Inducible nitric oxide synthase
COX-2: Cyclooxygenase-2
iCAM-1: Intercellular adhesion molecule-1
DAI: Disease activity index
RT-PCR: Real-time polymerase chain reaction
OTU: Operational taxonomic unit
PCA: Principal component analysis
PLS-DA: Partial least squares-discriminant analysis
NOR: Normal
PCA: Principal component analysis
TMG: Trimethylguanosine
HPA: Hydroxyhippuric acid
PBG: Phenylbutyrylglycine
HPAG: Hydroxyphenylacetylglycine
DPG: Dehydropregnenolone glucuronide
HPPG: Hydroxyphenylpropionylglycine
EG: Estriol-glucuronide
PAC: Phenylacetyl-carnitine
5-HTP: 5-Hydroxy-tryptophan
5-HT: 5-Hydroxytryptamine
ICAG: 3-Indole carboxylic acid glucuronide
GUS: β-Glucuronidase
F/B: *Firmicutes/Bacteroidetes*
SCFA: Short-chain fatty acid
UGT: Uridine 5′-diphospho-glucuronosyltransferase
