# Correction: Understanding the tonifying and the detoxifying properties of Chinese medicines from their impacts on gut microbiota and host metabolism: a case study with four medicinal herbs in experimental colitis rat model

**DOI:** 10.1186/s13020-023-00821-w

**Published:** 2023-08-30

**Authors:** Ting Li, Xuejiao Gao, Zhixiang Yan, Tai-Seng Wai, Wei Yang, Junru Chen, Ru Yan

**Affiliations:** 1 State Key Laboratory of Quality Research in Chinese Medicine, Institute of Chinese Medical Sciences, University of Macau, Taipa, Macao China; 2 Zhuhai UM Science & Technology Research Institute, Zhuhai, 519080 China

**Correction: Chinese Medicine (2022) 17:118 **10.1186/s13020-022-00673-w

Following publication of the original article [1], the authors reported an error in the Funding section and it needed to be updated.

The original version was:

This work was financially supported by the Science and Technology Development Fund of Macao SAR (043/2011/A2, 029/2015/A1), the National Natural Science Foundation (Ref. no. 81473281), and University of Macau (MYRG2015-00220-ICMS-QRCM).

The updated version is:

This work was financially supported by the Science and Technology Development Fund of Macao SAR (0091/2021/A2, 043/2011/A2, 029/2015/A1), Shenzhen-Hong Kong-Macau Science and Technology Program Category C (SGDX20210823103805038), the National Natural Science Foundation (Ref. no. 81,473,281), and University of Macau (MYRG2015 00220 ICMS QRCM).

The original article [1] has been corrected.
