# Supplementary material for: Understanding the tonifying and the detoxifying properties of Chinese medicines from their impacts on gut microbiota and host metabolism: a case study with four medicinal herbs in experimental colitis rat model
Source: Chin Med. 2022 Oct 4;17:118. doi: 10.1186/s13020-022-00673-w (PMC9533630; doi:10.1186/s13020-022-00673-w)
Supplement: Supplementary file 1 — Additional file 1. Additional methods. Figure S1. Hierarchical analysis of gut microbial composition on day 5 and day 7. Figure S2. PLS-DA of bacterial OTUs, genus, and host urinary metabolome on days 3, 5, 7. Figure S3. Correlation analysis between PC1s of PLSDA plot of bacterial OTUs and urinary metabolome on day 3, 5 and 7. Figure S4. Correlation analysis between A genus Rhodococcus and the metabolites β-alanine, guanine and creatinine; B genus Akkermansia and Parasutterella and the metabolites hydroxyphenylacetylglycine (HPAG) and phenylbutyrylglycine (PBG); C genus Acetatifactor and estriol-glucuronide (EG). Table S1. MRM ion pairs and calibration curves for quantitative analysis of main anthraquinones and the contents in RB. Table S2. Wavelengths and calibration curves for quantitative analysis of main flavonoids and the contents in SR. Table S3. MRM ion pairs and calibration curves for quantitative analysis of main components and their contents in Astragali Radix extract. Table S4. MRM ion pairs and calibration curves for quantitative analysis of ginsenosides and their contents in ginseng extract. [file 13020_2022_673_MOESM1_ESM.docx]

**Additional file 1:**

**Understanding the tonifying and the detoxifying properties of Chinese medicines from their impacts on gut microbiota and host metabolism: a case study with four medicinal herbs in experimental colitis rat model**

Ting Li^1,2†^, Xuejiao Gao^1,2†^, Zhixiang Yan^1,2^, Tai-Seng Wai^1,2^, Wei Yang^1,2^, Junru Chen^1,2^, Ru Yan^1,2,*^

^1^ State Key Laboratory of Quality Research in Chinese Medicine, Institute of Chinese Medical Sciences, University of Macau, Taipa, Macao, China

^2^ Zhuhai UM Science & Technology Research Institute, Zhuhai 519080, China

^†^ The authors contributed equally to this work.

**Preparation of herbal extracts and determination of main ingredients**

**Scutellaria Radix extract (SR)** Smashed powder (300 g) of Scutellaria Radix was extracted twice with 10 volumes of boiling water for 1 h. Then the supernatants were filtrated, combined, and concentrated using a rotary evaporator, and lyophilized to obtain 131.1 g extract (SR). Quantitative analysis of SR was completed on an Agilent 1200 HPLC system (Agilent Technologies, Santa Clara, CA, USA) equipped with a diode array detector. Sample separation was performed on an Agilent ZORBAX SB-C18 column (4.6×250 mm, 5 μm). The mobile phases consisted of water (A) and acetonitrile (B) both containing 0.1% formic acid and a gradient elution was adopted as follows: 0-20 min, 20%-25% B; 20-35 min, 25%-30% B; 35-40 min, 30-40% B; 40-50 min, 40% B; 50-55 min, 40-100% B; 55-60 min, 100% B. 60-61 min, 20% B. The column temperature, sampler room temperature, flow rate, and injection volume were set at 25°C, 4°C, 1 mL/min, and 10 μL, respectively. Formononetin was used as the internal standard. The detection wavelength was 288 nm and the results for the main flavonoids were shown in Table S2.

**Rhubarb extract (RB)** Three hundred grams of smashed powder of rhubarb was macerated in 3 L ethanol (75%, v/v) for 1 h, followed by heating at 80°C for 2 h under reflux at 150 mPa. The solvent was decanted and the extraction was repeated twice. The filtrates were combined, concentrated under reduced pressure, and lyophilized to obtain 170.8 g dark brown extract (RB). RB was quantified by HPLC-MS/MS method on a 4000 QTRAP MS System combined with Agilent 1200 HPLC system. The MS system utilizing electrospray ionization (ESI) interface was operated in positive mode. The chromatographic separation was achieved with an Agilent ZORBAX Eclipse XDB-C18 column (4.6×150 mm, 5 μm) on Agilent 1200 HPLC system coupled with 4000 QTRAP MS System. The mobile phase consisted of (A) water and (B) acetonitrile both containing 0.1% formic acid. The elution condition was optimized as follows: 10-33% B (0-10 min), 33-35% B (10-15 min), 35-40% B (15-20 min), 40-40% B (20-23 min), 40–60% B (23-25 min), 60-100% B (25-35 min), 100-00% B (35-40 min), and maintained at 10% B for 5 min. The injection volume was 2 μL and the flow rate was 0.5 mL/min. The temperature for column and auto-sampler were kept at 25°C and 4°C, respectively. 1,8-dioxyanthraquinone was used as the internal standard. The detected ion pairs and results for main anthraquinones were shown in Table S1.

**Astragali Radix extract (AR)** The Astragali Radix decoction (AR) was prepared as follows: smashed dry powder (300 g) was extracted with 2 L boiling water for 1 h, filtrated, and then the procedure was repeated twice. The combined filtrates were concentrated under reduced pressure and lyophilized to obtain AR (96.7 g). AR was dissolved by DMSO and diluted to 10 mg/ml by MeOH, then quantified by HPLC-MS/MS method on the 4000 QTRAP MS System. The chromatographic separation was performed by Agilent ZORBAX Eclipse XDB-C18 column (4.6×150 mm, 5 μm). The mobile phase consisted of (A) 0.1% formic acid in water and (B) acetonitrile containing 0.1% formic acid. The elution condition was optimized as follows: 35-65% B (0-8 min), 65-90% B (8-11 min), 90-90% B (11-19 min), 90-35% B (19-20 min) and equivalent at 35% B for 5 min. The injection volume was 5 μL and the flow rate was 0.5 mL/min. The temperature for column and auto-sampler were kept at 35 °C and 4 °C, respectively. The detected ion pairs and results for each compound were shown in Table S3.

**Ginseng extract (GS)**  Dried slices (500 g) of ginseng were refluxed with 15-fold distilled water at 100 °C for 1.5 h and filtered. The same process was repeated once, and the filtrates were combined and condensed to yield 371.4 g ginseng decoction (GS) under reduced pressure. GS was dissolved and diluted to 10 mg/ml by MeOH, then quantified by HPLC-MS/MS method on the forementioned MS System operated in positive mode. The chromatographic separation was performed by Agilent ZORBAX C18 column (2.1 mm × 100 mm, 1.8 µm). The mobile phase consisted of (A) 0.1% formic acid in water and (B) methanol containing 0.1% formic acid. The elution condition was optimized as follows: 0-3 min, 5% B; 3-4 min, 5-35% B; 4-8 min, 35-70% B; 8-35 min, 70-100% B; 35-35.1 min, 100-5% B; 35.1-45 min, 5% B. The injection volume was 2 μL and the flow rate was 0.2 mL/min. The temperature for column and auto-sampler was kept at 45 °C and 4°C, respectively. The detected ion pairs and results for main ginsenosides were shown in Table S4.


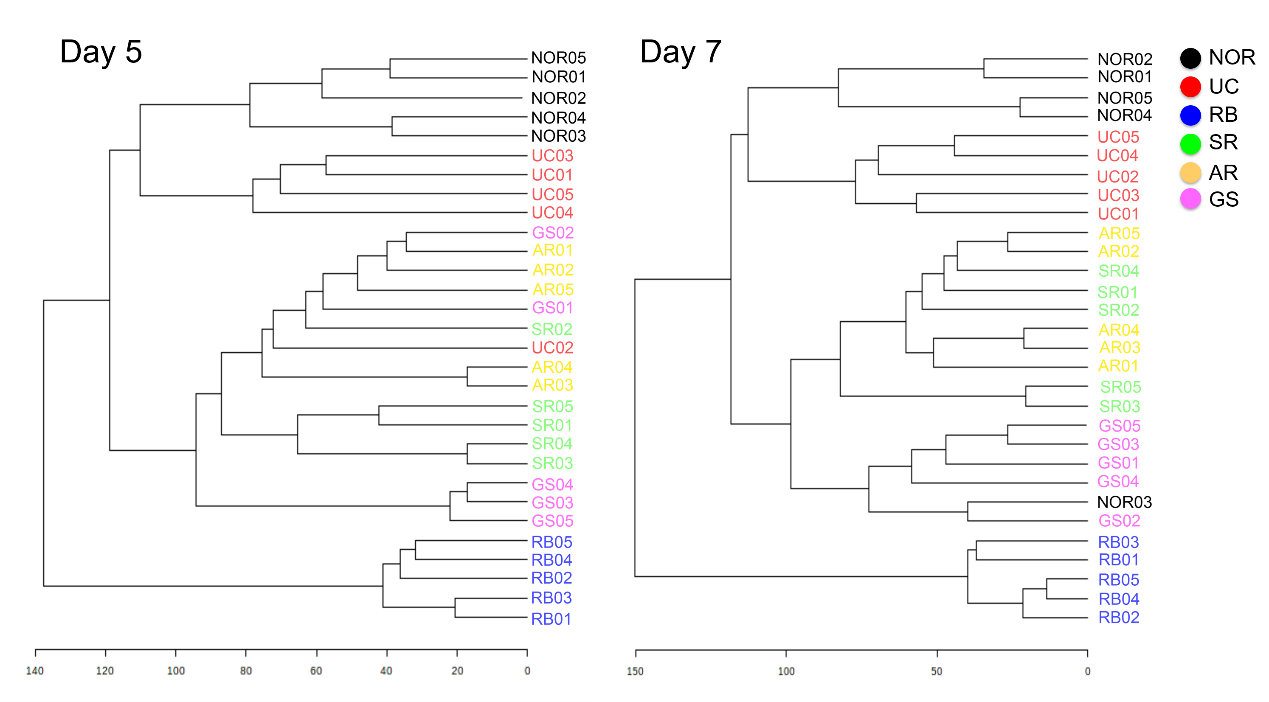
Figure S1 Hierarchical analysis of gut microbial composition on day 5 and day 7. Gut microbial composition in UC group was significantly different from that in NOR group, and RB treatment showed the most divergent gut microbiota.


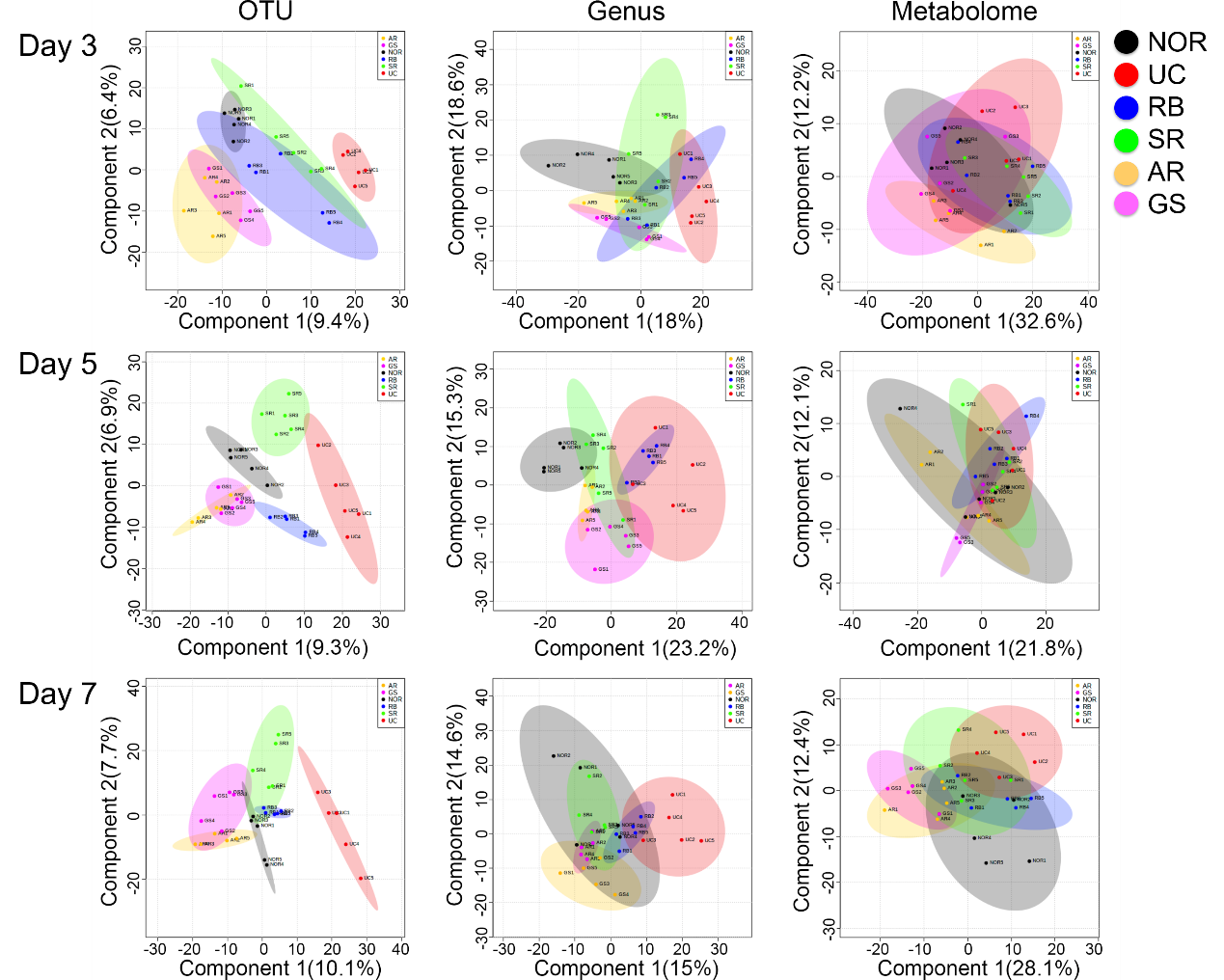


Figure S2 PLS-DA of bacterial OTUs, genus, and host urinary metabolome on days 3, 5, 7. Groups in the plot are shown with 95% confidence ellipses.


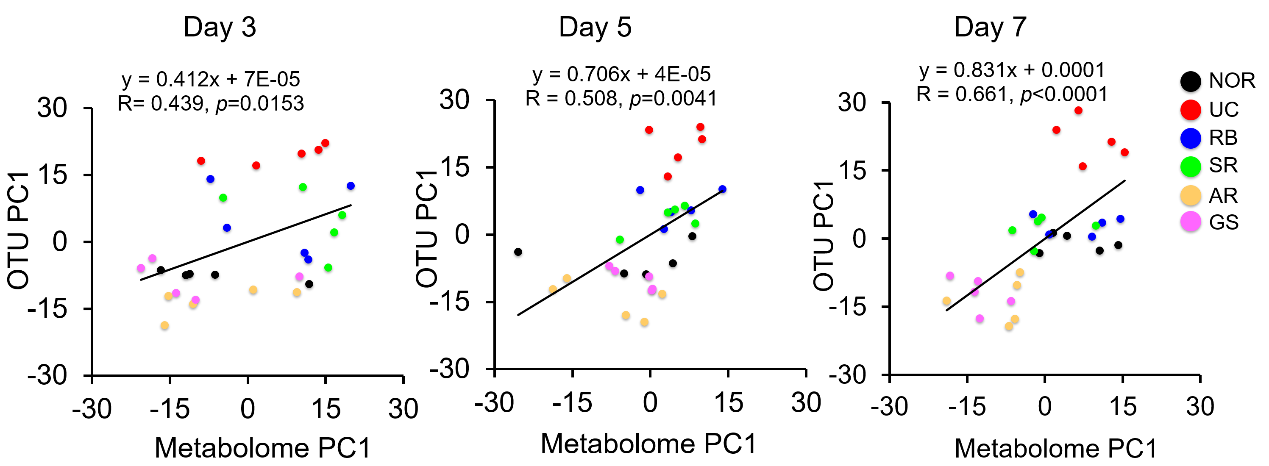


Figure S3 Correlation analysis between PC1s of PLSDA plot of bacterial OTUs and urinary metabolome on day 3, 5 and 7.


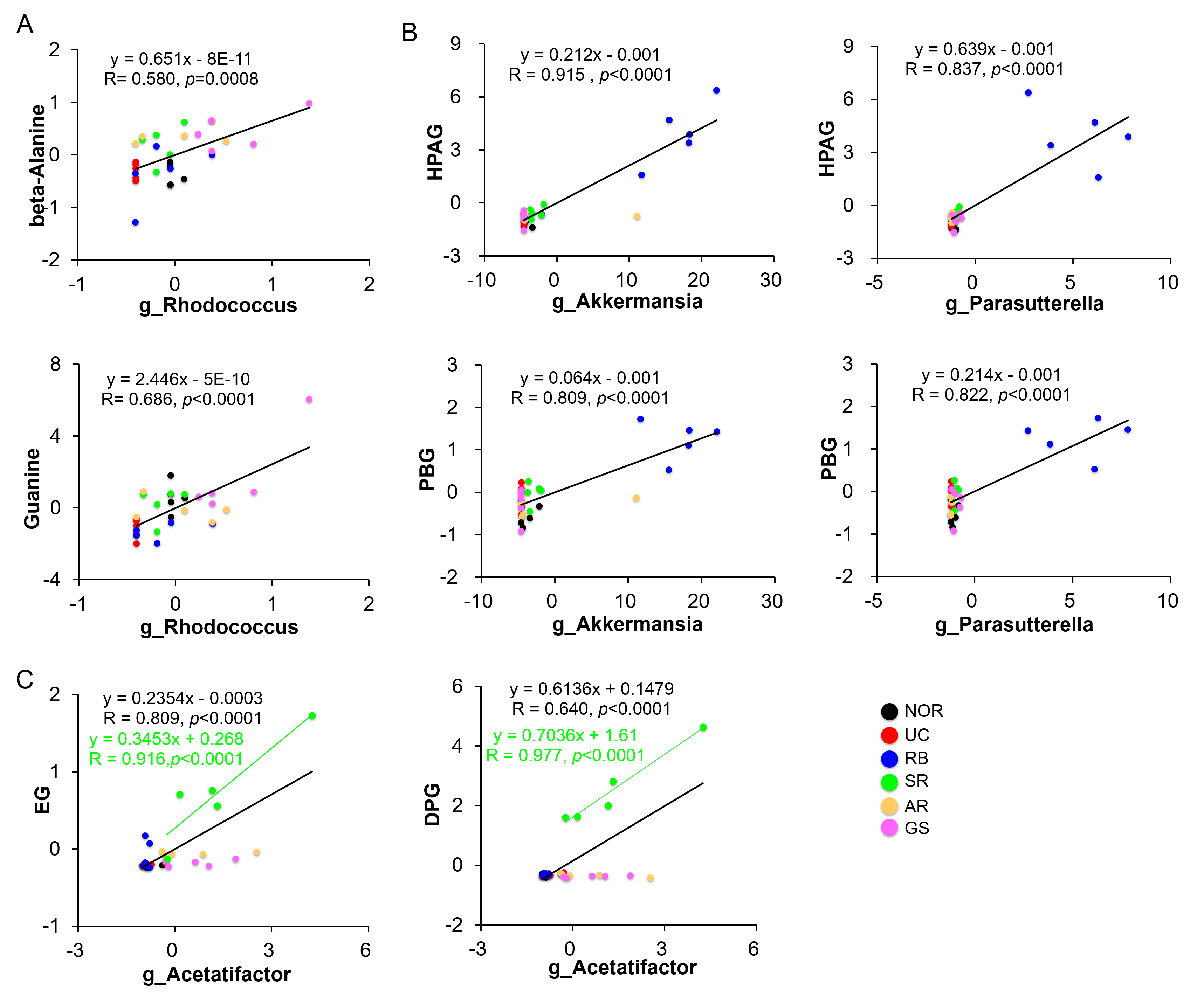


Figure S4 Correlation analysis between **(A)** gunus Rhodococcus and the metabolites β-alanine, guanine and creatinine; **(B)** genus Akkermansia and Parasutterella and the metabolites hydroxyphenylacetylglycine (HPAG) and phenylbutyrylglycine (PBG); **(C)** genus Acetatifactor and estriol-glucuronide (EG).

**Table S1** MRM ion pairs and calibration curves for quantitative analysis of main anthraquinones and the contents in RB.

| **Anthraquinone** | **MRM ion pair (m/z)** | **Concentration range (ng/mL)** | **Calibration curve equation** | **Correlation coefficient (r)** | **Contents in crude extract**  **(mg/100g extract)** |
| --- | --- | --- | --- | --- | --- |
| Emodin | 268.9/224.9 | 50~500 | y=0.0199x+0.715 | 0.9988 | 442.91 |
| Rhein | 283.1/238.9 | 50~5000 | y=0.00165x+0.00908 | 0.9999 | 1917.94 |
| Chrysophanol | 252.9/224.9 | 100~4000 | y=0.000193x+0.0202 | 0.9993 | 362.50 |
| Aleo emodin | 268.9/239.1 | 50~5000 | y=0.000056x-0.00107 | 0.9941 | 217.66 |
| Physicon | 268.9/224.9 | 200~4000 | y=0.000189x+0.0104 | 0.9983 | 406.99 |
| Emodin-glucoside | 431.0/269.0 | 25~500 | y=0.00496x+0.0177 | 0.9975 | 52.73 |
| Physicon-glucoside | 445.0/283.0 | 25~500 | y=0.000909x+0.00249 | 0.9980 | 41.19 |

**Table S2** Wavelengths and calibration curves for quantitative analysis of main flavonoids and the contents in SR.

| **Flavones** | **wavelength**  **(nm)** | **Concentration range**  **(μg/mL)** | **Calibration curve equation** | **Correlation coefficient (r)** | **Contents in crude extract**  **(mg/100g extract)** |
| --- | --- | --- | --- | --- | --- |
| Baicalin | 280 | 2.5~200 | y=21.059x-46.464 | 0.99974 | 199.24 |
| Oroxylin A-7-O-glucuronide | 280 | 2.5~200 | y=20.585x-15.662 | 0.9999 | 25.58 |
| Wogonoside | 280 | 2.5~200 | y=29.946x-24.972 | 0.9999 | 32.99 |
| Baicalein | 280 | 2.5~200 | y=31.589x-15.276 | 0.9999 | 15.64 |
| Oroxylin A | 280 | 2.5~200 | y=45.709x+0.9048 | 0.9999 | 4.40 |
| Wogonin | 280 | 2.5~200 | y=24.825x+3.2344 | 0.9999 | 2.48 |

**Table S3** MRM ion pairs and calibration curves for quantitative analysis of main components and their contents in Astragali Radix extract.

| **Components** | **MRM ion pair (m/z)** | **Concentration range (ng/mL)** | **Calibration curve equation** | **Correlation coefficient (r)** | **Contents in crude extract**  **(mg/100g extract)** |
| --- | --- | --- | --- | --- | --- |
| Astragaloside I | 869.3/143.1 | 200~4000 | y=0.0000656x+0.000186 | 0.9992 | 146.09 |
| Astragaloside II | 827.3/143.1 | 25~4000 | y=0.000142x+0.000984 | 0.9981 | 41.43 |
| Astragaloside III | 785.5/143.1 | 25~2000 | y=0.000163x-0.000386 | 0.999 | 29.23 |
| Astragaloside IV | 785.5/143.1 | 25~4000 | y=0.000125x+0.00111 | 0.999 | 32.44 |
| Formononetin | 269.0/253.0 | 25~500 | y=0.000438x+0.00436 | 0.9996 | 5.89 |
| Ononin | 431/269 | 25~1000 | y=0.00114x+0.00963 | 0.9983 | 26.93 |
| Calycosin | 285/270 | 50~1000 | y=0.000941x+0.0241 | 0.999 | 26.26 |
| Calycosin-glucoside | 447/285 | 50~5000 | y=0.000588x-0.0145 | 0.9974 | 101.62 |
| 7,2'-Dihydroxy-3',4'-  dimethoxyisoflavan | 303/123 | 25~2000 | y=0.000125x+0.000326 | 0.9988 | 13.41 |
| 3-Hydroxy-9,10-  dimethoxypterocarpan | 301/167 | 25~1000 | y=0.000909x+0.00249 | 0.9989 | 5.09 |

**Table S4** MRM ion pairs and calibration curves for quantitative analysis of ginsenosides and their contents in ginseng extract.

| **Ginsenosides** | **MRM ion pair (m/z)** | **Concentration range**  **(ng/mL)** | **Calibration curve equation** | **Correlation coefficient (r)** | **Contents in crude extract**  **(mg/100g extract)** |
| --- | --- | --- | --- | --- | --- |
| Re | 969.5/789.3 | 47.5~4350 | y=0.0000859x+0.000975 | 0.9964 | 17.98 |
| Rg1 | 823.5/643.5 | 160~4010 | y=0.000266x+0.00963 | 0.9984 | 38.29 |
| Rg2 | 807.5/807.5 | 39.3~3930 | y=0.000941x+0.0208 | 0.9994 | 84.40 |
| Rf | 824.5/365.5 | 78.5~7850 | y=0.0000382x+0.000365 | 0.9971 | 80.00 |
| Rh1 | 661.5/661.5 | 128~3190 | y=0.000271x+0.0131 | 0.9984 | 94.96 |
| F1 | 661.5/661.5 | 63.9~3190 | y=0.000298x+0.0038 | 0.9972 | 66.80 |
| PPT | 499.5/499.5 | 9.53~2380 | y=0.000281x-0.0000249 | 0.9994 | 0.81 |
| Rb1 | 1131.5/1131.5 | 55.5~5550 | y=0.0000708x-0.00013 | 0.9977 | 131.16 |
| Rc | 1101.5/789.5 | 10.8~5400 | y=0.000123x-0.000417 | 0.9997 | 33.30 |
| Rb2+Rb3 | 1101.5/789.5 | 10.8~5400 | y=0.00023x-0.000272 | 0.9996 | 14.83 |
| Rd | 969.5/969.5 | 9.47~4740 | y=0.000197x-0.0000914 | 0.9997 | 27.15 |
| F2+Rg3 | 807.5/807.5 | 78.5~7850 | y=0.000421x+0.00463 | 0.9988 | 272.24 |
| Rh2 | 645.5/645.5 | 31.1~3110 | y=0.000288x+0.00204 | 0.9991 | 6.26 |
| CK | 645.5/645.5 | 31.1~3110 | y=0.00385x-0.00062 | 0.9984 | 3.51 |
| PPD | 425.5/109.3 | 25~4000 | y=0.000125x+0.00111 | 0.999 | ND |

ND: not detectable

**Table S5** Primer sequences for PCR assay of target genes in the rat.

| **Target gene** | **Primer** | **Sequence 5’-3’** | **Reference** |
| --- | --- | --- | --- |
| IL-10 | Forward | CTG TCA TCG ATT TCT CCC CTG T | [[2](#_ENREF_2)] |
|  | Reverse | CAG TAG ATG CCG GGT GGT TC |  |
| TGF-β1 | Forward | CTT CAG CTC CAC AGA GAA CTG C |  |
|  | Reverse | CAC GAT CAT GTT GGA CAA CTG CTC C |  |
| IFN-γ | Forward | TCG CAC CTG ATC ACT AAC TTC TTC |  |
|  | Reverse | CGA CTC CTT TTC CGC TTC C |  |
| MCP-1 | Forward | CTA TGC AGG TCT GTC ACG CTT C |  |
|  | Reverse | CAG CCG ACT CAT TGG GAT CA |  |
| IL-4 | Forward | CAT CGG CAT TTT GAA CGA G | [[3](#_ENREF_3)] |
|  | Reverse | CGA GCT CAC TCT CTG TGG TG |  |
| IL-6 | Forward | CAC AAG TCC GGA GAG GAG AC | [[4](#_ENREF_4)] |
|  | Reverse | CAG AAT TGC CAT TGC ACA AC |  |
| IL-1β | Forward | CCT CTG CCA AGT CAG GTC TC |  |
|  | Reverse | GAA TGT GCC ACG GTT TTC TT |  |
| TNF-α | Forward | CTT CTG TCT ACT GAA CTT C | [[5](#_ENREF_5)] |
|  | Reverse | AAG ATG ATC TGA GTG TGA |  |
| iNOS | Forward | CTT TGC CAC GGA CGA GAC | [[6](#_ENREF_6)] |
|  | Reverse | TCA TTG TAC TCT GAG GGC TGA |  |
| COX-2 | Forward | TTA AAA TGA GAT TGT CCG AA |  |
|  | Reverse | AGA TCA CCT CTG CCT GAG TA |  |
| ICAM-1 | Forward | CGT GGC GTC CAT TTA CAC CT | [[7](#_ENREF_7)] |
|  | Reverse | TTA GGG CCT CCT CCT GAG C |  |
| β-actin | Forward | ATC GCT GAC AGG ATG CAG AA | [[8](#_ENREF_8)] |
|  | Reverse | TAG AGC CAC CAA TCC ACA CAG |  |

References:

[1] Jian-Qing Ruan; Shang Li; Ya-Ping Li; Wen-Jin Wu; Simon Ming-Yuen Lee; Ru Yan; The presystemic interplay between gut microbiota and orally administered calycosin-7-o-β-d-glucoside, Drug Metabolism and Disposition, 2015, 43: 1601-1611.

[2] Kavindra Kumara Wijesundera; Takeshi Izawa; Anusha Hemamali Tennakoon; Hiroshi Murakami; Hossain M Golbar; Chisa Katou-Ichikawa; Miyuu Tanaka; Mitsuru Kuwamura; Jyoji Yamate; M1-and m2-macrophage polarization in rat liver cirrhosis induced by thioacetamide (taa), focusing on iba1 and galectin-3, Experimental and molecular pathology, 2014, 96: 382-392.

[3] Kei Seno; Jun Ohno; Nobutaka Ota; Takao Hirofuji; Kunihisa Taniguchi; Lupus-like oral mucosal lesions in mercury-induced autoimmune response in brown norway rats, BMC immunology, 2013, 14: 1-10.

[4] Rong Zhou; Zailiang Yang; Xurong Tang; Yan Tan; Xiaofeng Wu; Feng Liu; Propofol protects against focal cerebral ischemia via inhibition of microglia-mediated proinflammatory cytokines in a rat model of experimental stroke, Plos One, 2013, 8: e82729.

[5] Tu Thi Ngoc Nguyen; Yong Min Kim; T Doohun Kim; Oanh Thi Tu Le; Jae Jin Kim; Ho Chul Kang; Hiroshi Hasegawa; Yasunori Kanaho; Ilo Jou; Sang Yoon Lee; Phosphatidylinositol 4-phosphate 5-kinase α facilitates toll-like receptor 4-mediated microglial inflammation through regulation of the toll/interleukin-1 receptor domain-containing adaptor protein (tirap) location, Journal of Biological Chemistry, 2013, 288: 5645-5659.

[6] Li Jiang; Fan Xu; Wenjing He; Lifei Chen; Haibin Zhong; Yu Wu; Siming Zeng; Li Li; Min Li; Cd200fc reduces tlr4-mediated inflammatory responses in lps-induced rat primary microglial cells via inhibition of the nf-κb pathway, Inflammation research, 2016, 65: 521-532.

[7] Shujuan Qiu; Guiling Sun; Yunxia Zhang; Xiangling Li; Rong Wang; Involvement of the nf-κb signaling pathway in the renoprotective effects of isorhamnetin in a type 2 diabetic rat model, Biomedical Reports, 2016, 4: 628-634.

[8] Huihui Liu; Bin Wu; Guoyu Pan; Lei He; Zhixiong Li; Mingsong Fan; Longhai Jian; Mingcang Chen; Ke Wang; Chenggang Huang; Metabolism and pharmacokinetics of mangiferin in conventional rats, pseudo-germ-free rats, and streptozotocin-induced diabetic rats, Drug Metabolism and Disposition, 2012, 40: 2109-2118.
